# Attenuation of IFN signaling due to m^6^A modification of the host epitranscriptome promotes EBV lytic reactivation

**DOI:** 10.1186/s12929-023-00911-9

**Published:** 2023-03-14

**Authors:** Dipayan Bose, Xiang Lin, Le Gao, Zhi Wei, Yonggang Pei, Erle S. Robertson

**Affiliations:** 1 Department of Otorhinolaryngology-Head and Neck Surgery, and Tumor Virology Program, Perelman School of Medicine, University of Pennsylvania, 19104 Philadelphia, PA USA; 2 Department of Computer Science, New Jersey Institute of Technology, 07102 New Jersey, United States of America; 3 School of Public Health and Emergency Management, Southern University of Science and Technology, 518055 Shenzhen, Guangdong China

**Keywords:** N6-methyladenosine, Epitranscriptome, Lytic reactivation, Innate Immune response, RNA immunoprecipitation

## Abstract

**Background:**

Reactivation of Epstein Barr virus (EBV) leads to modulation of the viral and cellular epitranscriptome. N6-methyladenosine (m^6^A) modification is a type of RNA modification that regulates metabolism of mRNAs. Previous reports demonstrated that m^6^A modification affects the stability and metabolism of EBV encoded mRNAs. However, the effect of reactivation on reprograming of the cellular mRNAs, and how this contributes to successful induction of lytic reactivation is not known.

**Methods:**

Methylated RNA immunoprecipitation sequencing (MeRIP-seq), transcriptomic RNA sequencing (RNA-seq) and RNA pull-down PCR were used to screen and validate differentially methylated targets. Western blotting, quantitative real-time PCR (RT-qPCR) and immunocytochemistry were used to investigate the expression and localization of different proteins. RNA stability and polysome analysis assays were used to detect the half-lives and translation efficiencies of downstream genes. Insertion of point mutation to disrupt the m^6^A methylation sites was used to verify the effect of m^6^A methylation on its stability and expression levels.

**Results:**

We report that during EBV reactivation the m^6^A eraser ALKBH5 is significantly downregulated leading to enhanced methylation of the cellular transcripts DTX4 and TYK2, that results in degradation of TYK2 mRNAs and higher efficiency of translation of DTX4 mRNAs. This resulted in attenuation of IFN signaling that promoted progression of viral lytic replication. Furthermore, inhibition of m^6^A methylation of these transcripts led to increased production of IFN, and a substantial reduction in viral copy number, which suggests abrogation of lytic viral replication.

**Conclusion:**

Our findings illuminate the significance of m^6^A modification in overcoming the innate immune response during EBV reactivation. We now report that during lytic reactivation EBV targets the RNA methylation system of the host to attenuate the innate immune response by suppressing the interferon signaling which facilitates successful lytic replication of the virus.

**Supplementary Information:**

The online version contains supplementary material available at 10.1186/s12929-023-00911-9.

## Background

Modulation of the cellular epitransciptome plays an important role in regulation of gene expression. Recent studies have clearly demonstrated that different posttranscriptional modifiers can function as important players in different pathophysiological conditions. N6-methyladenosine (m^6^A) is the most abundant internal mRNA modification in eukaryotes that is also reversible. It is involved in regulation of RNA metabolism, folding, export, splicing, and translation [1]. Many studies have focused not only on the role of m^6^A modification in different viral infections including EBV [2–7], but also on how m^6^A modification may be associated with the innate immune anti-viral response through IFN signaling [8–11]. Therefore, m^6^A modification is emerging as an important field of research in the context of host pathogen interaction.

Type1 IFN response is the key regulator of innate antiviral response that involves IFN-α/β receptor (IFNAR)[12, 13], and the signaling cascade of the JAK-STAT pathway, which results in transcriptional initiation of other IFN responsive genes [14]. Dysregulation of this IFN signaling leads to an abrogated anti-viral response [15]. Stringent control of the type I interferon signaling pathway is therefore important for maintaining host immune responses and homeostasis, yet the molecular mechanisms responsible for its tight regulation are still poorly understood. Thus, tight regulation at the post-transcription and post-translational levels are critical for establishing cellular homeostasis.

Epstein-Barr virus (EBV or HHV4) is a member of the herpes virus family that accounts for approximately 1.5% of the total number of cancers annually [16, 17]. EBV, like many of the herpesvirus family members has two distinct phases of infection namely latent and lytic infection [18]. EBV has a wide range of host cells but primarily infects B cells and epithelial cells. Different viruses have evolved unique strategies to tackle and bypass the host defense mechanism for establishment in susceptible hosts. In human primary B-cells, EBV normally establishes latent infection which can be reactivated to drive lytic replication and progeny production [19]. Reactivation can be induced by different strategies which include chemical induction, UV irradiation, and induction of epigenetic modifiers and others. During the initial phase of lytic reactivation, the virus subverts the host innate immune responses, and replicates within the host cells using the host cell machinery. Previous studies on how EBV escapes the host defense system during its lytic reactivation are mainly focused on the viral encoded microRNAs [20, 21], or different viral-encoded genes that interact with cellular genes to shut off the host immune responses [22].

Pathogen-associated molecular pattern (PAMPs) recognizes foreign nucleic acids, triggers the activation of type I interferons and the inflammasome signaling pathway [23]. This leads to production of proinflammatory cytokines and induction of subsequent adaptive immune responses. The cellular sensors and their subsequent adapters require the kinase TBK1 to activate the transcription factor IRF3 [24], which leads to induction of type I interferon signaling [25]. DTX4 is an E3 Ubiquitin Ligase that targets TBK1 and leads to its degradation [26]. TBK1 positively regulates type I interferon signaling through IRF3 phosphorylation that leads to its activation which is important for host immune response to viral replication [26]. IFN functions in an autocrine as well as paracrine manner and binds to the IFN receptors. At the cytoplasmic tail of the IFN receptors, the Tyrosine kinase 2 (TYK2), adapter kinase molecules get auto-phosphorylated and triggers activation of STAT signaling, which in turn activates more of the IFN responsive genes thus intensifying the antiviral response [27]. Therefore, both TYK2 and DTX4 are important to fully activate the innate immune antiviral response.

Our group has previously shown that EBV regulates the host m^6^A modifying machinery to modify virus encoded mRNAs, which were post transcriptionally modified resulting in altered expression of the viral genes [28]. EBV encoded nuclear antigen 3 C (EBNA3C) was identified as the main antigen that hijacks the methyltransferase system of the host by interacting with the METTL14 reader molecule which affects stability of viral mRNAs. In the present study, we were interested in investigating the host epitranscriptome to identify the most abundant mRNAs that were differentially modified during EBV lytic reactivation. Our results identified several mRNAs that were uniquely methylated during lytic reactivation when compared to latent infection. From these genes we focused on two genes, DTX4 and TYK2, and correlated their methylation status to their function linked to the interferon response. Thus, we have now elucidated a novel mechanism by which EBV can subvert the host IFN response during the early lytic reactivation resulting in enhanced viral copy numbers and successful viral replication.

## Methods

### Cell lines, plasmid vectors, transfection, and lentivirus transduction

BJAB, Akata-EBV, and BL41-B95.8 cell lines were kindly provided by Elliott Kieff (Harvard Medical School, Boston, MA). LCL were generated in our laboratory. HEK-293 (human embryonic kidney cell line) were provided by Jon Aster (Brigham and Woman’s Hospital, Boston, MA). All B cells were maintained in RPMI medium supplemented with 10% bovine growth serum (BGS) with antibiotics. HEK293 cells were maintained in DMEM medium with 5% BGS and antibiotics. Constructs for Flag-tagged METTL14 and Myc-tagged DTX4 constructs were described earlier [29]. Point mutations were inserted in the Myc-tagged DTX4 gene by following the protocol previously described [30]. Transfections were carried out using jetPRIME reagent (Polyplus Transfection Inc, New York, NY) or by electroporation. ShRNAs targeting DTX4, TYK2, ALKBH5 and YTHDF2 were generated using the pGIPZ vector [31].

For production of lentivirus, pGIPZ clones along with packaging and helper plasmids were transfected into HEK293T cells using HBS. Lentivirus transduction was performed in a total volume of 1 ml in the presence of 20 µg/ml Polybrene. Unless otherwise stated, all cultures were incubated at 37 °C in a humidified chamber with 5% CO_2_. EBV positive cells were induced to lytic reactivation by using TPA (20 ng/ml) and Butyric acid (3 mM) for 48 h induction.

### RNA immune precipitation (RIP) and preparation for sequencing and PCR

Isolation of m^6^A-containing mRNA fragments was performed as previously described with minor modifications [28]. Total RNA was extracted from cells using Trizol Reagent (Invitrogen, Inc., Carlsbad, CA) and fragmented in a buffer containing 100 mM Tris-HCl at pH 8.0 and 100 mM ZnCl_2_ followed by incubation at 94 °C for 8 min. Post fragmentation, the mRNAs with sizes close to 120 nucleotides was validated using a BioRad Geldoc Station (Bio-Rad, Hercules, CA). Before immunoprecipitation, 10–15 µg of anti-m^6^A antibody was incubated with Pierce Protein-A Agarose beads slurry (Thermo Fisher Scientific, Waltham, MA) in 250 µl PBS with 0.5% BSA at 4 °C for 2 h. The beads were washed three times in cold PBS with 0.5% BSA. Total 900 µg of fragmented RNA was added to the antibody-bound beads in 250 µl IP buffer supplemented with RNase inhibitor (Promega Inc, Madison, WI), and the mixture was mixed at 4 °C for 2 h. The beads were washed with 1 ml IP buffer (50 mM Tris-HCL pH 7.5, 750 mM NaCl and 0.5% NP40) 4 times before eluted with 100 µl IP buffer supplemented with 6.67 mM of m^6^A salt (M2780, Sigma-Aldrich, St. Louis, MO). The mixture was incubated for 1 h at 4 °C with continuous shaking and the eluate collected. A second elution was carried out and the eluates were pooled together before purification with 2.5-fold ethanol and 1/10 volume of Sodium acetate solution pH 5.2. RNA was washed with 75% ethanol and dissolved in RNase free H_2_O.

Post immune precipitation of the RNA, the samples were either sequenced or used for Real time PCR using specific primers. For sequencing and data analysis the samples were processed as described previously [28].

### Data analysis for MeRIP-sequencing sets

We checked the sequencing quality by using the FastQC tool. Since all bases in our reads have quality scores higher than 30 and the adapters were not detected, we skipped the trimming step. After quality check, the RIP-seq reads were aligned, sorted, and transformed into Bam files using STAR [32]. The alignment used Human reference genome with the annotation of the splice sites (Human Genome Assembly GRCh38.p13).

The peak calling was performed using MACS3 [32, 33]. For sequencing duplicate samples were processed at the same time and were sequenced. The normalized replicate samples sequencing data were averaged by Convfuzze and presented for easy understanding. The genome sequences of peaks were extracted from BED files from Human reference genome using BED files from MACS3 by BEDTools [34]. The motifs were analyzed by using the peak sequences and online based software STREME [35]. For coverage plots, BED files for TYK2 and DTX4 were extracted from the Gene transfer format (gtf) annotation file and the plots were created by Covfuzze (https://github.com/al-mcintyre/CovFuzze/blob/master/covfuzze/covfuzze.py). The coverage plot was represented as normalized coverage where normalized coverage = (read count × read length)/total genome size.

### Generation of site-specific point mutation

Point mutations were inserted in the Myc-tagged DTX4 gene clone by the following protocol. Forward and reverse primers were designed with 20 nucleotides complementary to each other on both the sides of the point mutation (Supplementary Table 1) using both forward and reverse primers in the PCR reaction for 12–18 cycles. The PCR products were denatured, and then reannealed. The non-mutated methylated parental plasmid was digested with DpnI and the remaining plasmids were transformed into E. coli cells.

### Western blot analysis

Cell lysates were prepared from a minimum of 10 million cells, and total protein were harvested. 40–100 µg of protein for each sample was loaded in each well and resolved on SDS-polyacrylamide gels followed by wet transfer to nitrocellulose membrane. 5% skimmed milk or BSA was used for blocking at room temperature for 1 h with gentle shaking. The membranes were incubated with primary antibodies overnight at 4 °C followed by 1 h washing with TBST and probing with infrared (IR) dye conjugated secondary antibody. Membranes were scanned using an Odyssey scanner (LI-COR Inc., Lincoln, NE) for detection of specific antigens and the band intensities were measured using the ImageQuant, Odyssey software. The band intensities were normalized with that of the loading control (GAPDH) and the fold intensity values were represented below each blot. The antibodies used are listed with their suppliers in Supplementary Table 2.

### RNA isolation, immunoprecipitation (RIP) and real time PCR

Total RNAs were extracted using Trizol reagent (Invitrogen, Inc., Carlsbad, CA) and treated with DNase I (Invitrogen, Inc., Carlsbad, CA). The cDNAs were prepared with Superscript II reverse transcriptase kit (Invitrogen, Inc., Carlsbad, CA) according to manufacturer’s protocol.

Copy number calculations from EBV preparation were performed using the standard method with minor modifications [36]. The primer sequences used are listed in Supplementary Table 3. Known copy number of EBNA1 encoding plasmid was used to generate the standard curve. The total DNA was isolated from 20 million cells and resuspended in 20 µl of TE buffer. 1 µl of the prepared samples was used for quantification.

For RNA immunoprecipitation, total RNA was extracted from cells using Trizol Reagent (Invitrogen, Inc., Carlsbad, CA) and fragmented in a buffer containing 100 mM Tris-HCl at pH 8.0 and 100 mM ZnCl_2_ followed by incubation at 94 °C for 8 min. Post fragmentation, the mRNAs with sizes close to 120 nucleotides was validated using a BioRad Geldoc Station (Bio-Rad, Hercules, CA). Before immunoprecipitation, 10–15 µg of anti-m^6^A antibody was incubated with Pierce Protein-A Agarose beads slurry (Thermo Fisher Scientific, Waltham, MA) in 250 µl PBS with 0.5% BSA at 4 °C for 2 h. The beads were washed three times in cold PBS with 0.5% BSA. Total 900 µg of fragmented RNA was added to the antibody-bound beads in 250 µl IP buffer supplemented with RNase inhibitor (Promega Inc, Madison, WI), and the mixture was mixed at 4 °C for 2 h. The beads were washed with 1 ml IP buffer (50 mM Tris-HCL pH 7.5, 750 mM NaCl and 0.5% NP40) 4 times before eluted with 100 µl IP buffer supplemented with 6.67 mM of m^6^A salt (M2780, Sigma-Aldrich, St. Louis, MO). The mixture was incubated for 1 h at 4 °C with continuous shaking and the eluate collected. A second elution was carried out and the eluates were pooled together before purification with 2.5-fold ethanol and 1/10 volume of Sodium acetate solution pH 5.2. RNA was washed with 75% ethanol and dissolved in RNase free H_2_O.

Post immune precipitation of the RNA, the samples were either sequenced or used for Real time PCR using specific primers.

The quantitative real-time PCR analyses were performed by using SYBR green real-time master mix (MJ Research Inc., Waltham, MA). The real time data were analyzed for fold change based on the internal control GAPDH or 18 S as a housekeeping gene. For the MeRIP-PCR, the fold enrichment was calculated and normalized based on the signal obtained from the IgG control signal.

### Cell fractionation

To investigate the transport of mRNA from the cytosol to nucleus, we performed cellular fractionation as previously performed with few modifications [37]. 30 million cells were harvested and lysed in 200 µL lysis buffer (10mM Tris-HCl [pH 7.4], 140 mM NaCl, 1.5 mM MgCl_2_, 10 mM EDTA, 0.5% NP-40) on ice for 5 min. Following centrifugation at 12,000xg at 4˚C for 5 min, the supernatant (cytoplasmic fraction) was collected, and the nuclear pellet was rinsed twice with lysis buffer. RNA was extracted from cytoplasmic and nuclear pellets separately using Trizol reagent and analyzed by qRT-PCR using specific primers.

### Measurement of RNA stability

20 million LCL and Akata cells were cultured and reactivated using TPA and Butyric acid or mock reactivated using PBS. After 12 and 24 h post reactivation 1 µM Actinomycin D (Sigma-Aldrich, St. Louis, MO, USA) were added to the medium. RNA was extracted using Trizol reagent 36 h post reactivation and analyzed by rtPCR using specific primers. Data were normalized relative to the Actinomycin untreated (mock) and unreactivated cells with each time point after treatment [37].

### Polysome profiling

EBV positive B cells (LCL and Akata) were cultured, reactivated, and pulsed with cycloheximide (0.2 mM; Sigma-Aldrich, St. Louis, USA) for 10 min and were lysed by cytoplasmic lysis buffer (200 mM KCl, 25 mM HEPES pH 7.0, 10 mM MgCl_2_, 2% n-Dodecyl b-D-maltoside (DDM; Chem-Impex), 0.2 mM cycloheximide (Sigma-Aldrich), 1 mM DTT, 40 U RNaseIn) for 15 min on ice. The lysates were ultracentrifuged by loading on 15–50% sucrose gradients prepared in polysome gradient buffer (200 mM KCl, 25 mM HEPES pH 7.0, 15 mM MgCl_2_, 1 mM DTT, 0.2 mM cycloheximide) at 35,000xg for 4 h at 4˚C. Following ultracentrifugation, 16 fractions were collected from each sample. RNA was extracted from each fraction using Trizol (Thermo Fisher, Waltham, MA), and RNA quality was checked on a 1% agarose gel, reverse transcribed and qRT-PCR were performed using specific primers for TYK2 and DTX4 genes.

### Immunofluorescence

For immunofluorescence, cells were seeded on glass coverslips in 24-well plates. After treatment, cells were fixed with 4% paraformaldehyde at 4 °C for 60 min and permeated with 0.2% Triton X-100 in PBS for 10 min. Cells were stained with IRF9 antibodies (1:200), and counter stained with Alexa594 conjugated secondary antibody (1:400). Nuclei were visualized by staining with DAPI for 2 min. Images were acquired using a Fluoview FV300 confocal microscope and the images were analyzed using ImageJ software for image analysis.

### Statistical analysis

The experiments were performed in triplicates and the mean scores were examined by using Student’s t-test. All statistical tests were performed using either Microsoft Office Excel or by GraphPad Prism 8 software (San Diego, CA, USA). A P-value of 0.05 was considered to be a statistically significant difference. A P-value of 0.01 was considered to indicate high statistical significance.

## Results

### EBV lytic reactivation remodels m^6^A modification of cellular transcripts

**Fig. 1:**
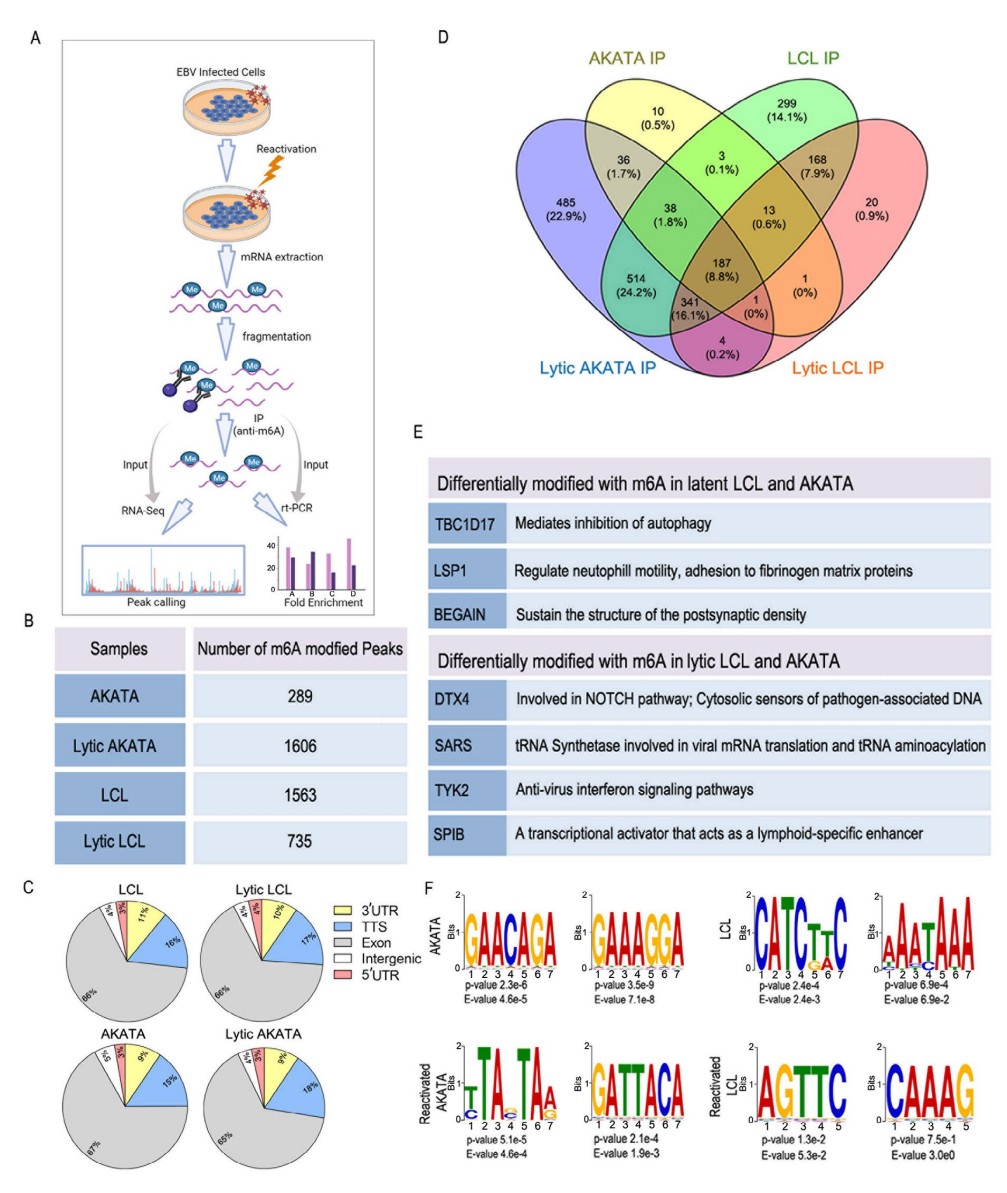
Lytic reactivation of EBV alters m^6^A Modification of host cell transcripts (**A**) Graphical representation of the work-flow. (**B**) Number of differentially modified peaks in Akata and LCL during latency and lytic reactivation. (**C**) Distribution of m^6^A peaks in four topological regions of cellular RNAs during latency and lytic EBV reactivation. UTR, untranslated region. TTS, Translation termination site. (**D**) Venn diagram comparing the number of methylated human genes before (latent) and after induction for lytic reactivation in LCL and Akata cells. (**E**) List of genes that were uniquely methylated during latency and during lytic reactivation in Akata and LCL cells and their corresponding cellular functions. (**F**) Most significant motifs in cellular m^6^A peaks identified by STREME (Sensitive, Thorough, Rapid, Enriched Motif Elicitation) [35] in latent and reactivated LCL and Akata cells. The top two motifs with the smallest p- values has been listed

Lytic reactivation of the EBV-infected cells changes the expression of pro-viral and antiviral genes products. m^6^A methylation has been linked to RNA metabolism which includes the efficiency of translation, as well as the stability and transport of the transcripts. These changes lead to altered expression of the encoded proteins. We hypothesized that altered m^6^A modification would influence the expression of host genes to regulate viral lytic reactivation. We therefore measured changes in m^6^A modification of host transcripts during lytic reactivation by using methylated RNA immunoprecipitation followed by sequencing and rtPCR (Fig. 1A). We performed MeRIP-seq on RNA isolated from EBV positive LCL, and Akata cells under both latent and reactivated conditions. We identified different numbers of methylated peaks in each of the cell types during latency and lytic reactivation ranging from hundreds to over 1600 peaks (Fig. 1B). Based on their positions within mRNA, we classified these m^6^A sites into five regions: 5′ UTR, Exonic, 3′ UTR, Intergenic and Translational start site (TTS). However, there was no significant changes in the distribution from latent to lytic reactivation (Fig. 1C). We further analyzed the differential methylation of genes between latent and lytic replication and found a total of 7 different cellular mRNAs that were differentially methylated during lytic reactivation (4 mRNAs) when compared to latent (3 mRNAs) in both LCL and Akata (Fig. 1D and E). The methyltransferase enzymes recognize conserved motifs on the mRNAs, and the writer complex then incorporates a methyl group on the adenosine residue of that motif [38]. We further wanted to analyze the sequences involved and identified the specific motifs that were methylated (Fig. 1F).

Functional annotation of the m^6^A-methylated genes in reactivated samples revealed an enrichment of genes with roles linked to a number of different cellular processes including interferon signaling, cellular growth, antigen processing and presentation (Suppl. Figure 1 A). Furthermore, we investigated 26 genes that were uniquely methylated in reactivated LCL compared to latent, and 27 uniquely methylated genes in latent Akata compared to lytic Akata (p < 0.05 and fold change either > 2 or less < 2). We performed a pathway analysis with these genes separately and identified both the TYK2 and DTX4 genes that were hypermethylated in lytic EBV positive cells when compared to latent cells (Suppl. Figure 1B, C) and are two genes that are key players in the IFN signaling pathway. Previous reports suggested that during lytic reactivation, IFN signaling was differentially regulated and that different viral antigens can play crucial roles in its regulation [39–41]. Our findings now suggest that differential methylation of the cellular mRNAs involved in this pathway can control the IFN signaling response due to reactivation of EBV.

### EBV lytic reactivation induces hypermethylation of TYK2 and DTX4 mRNAs

**Fig. 2:**
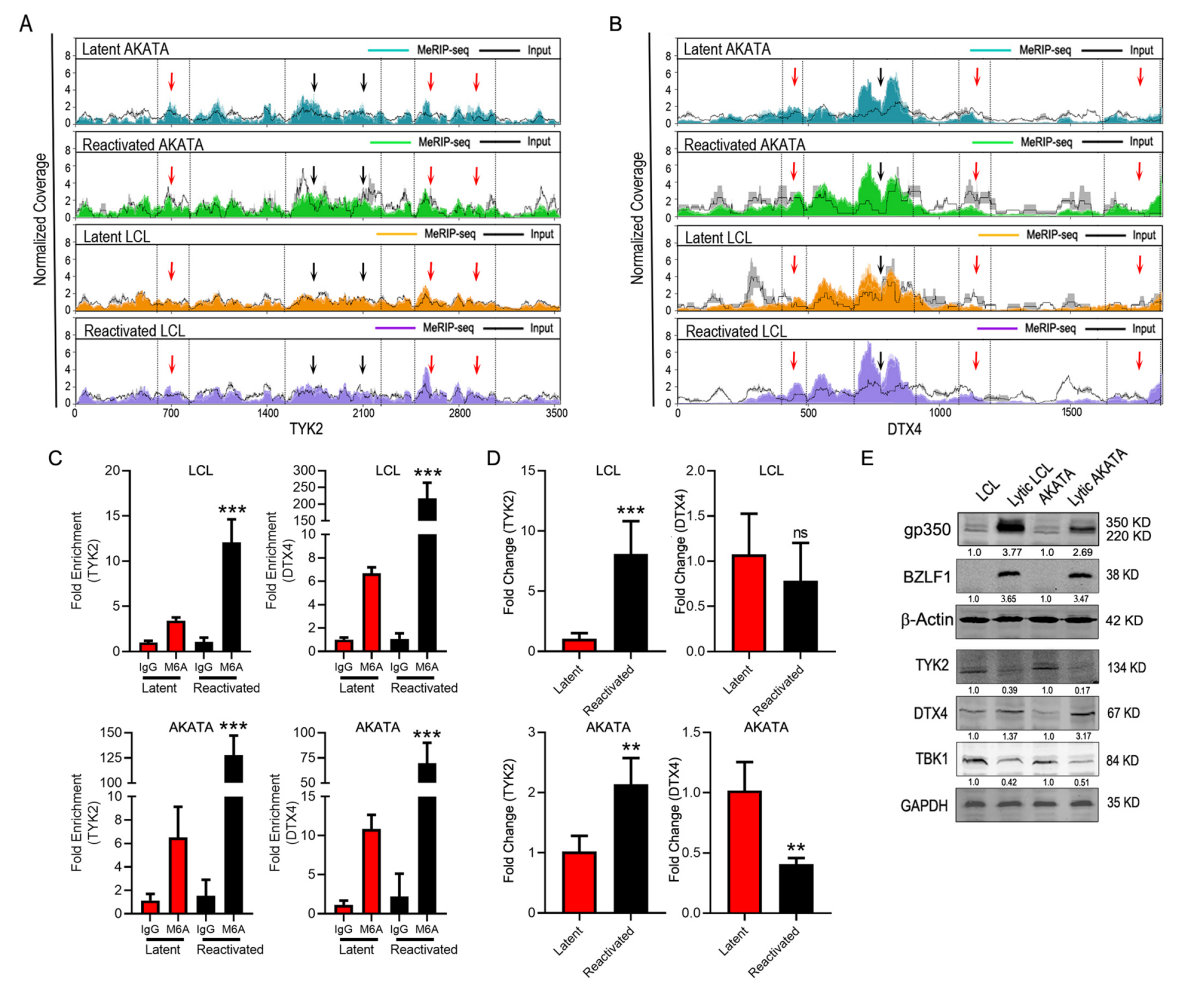
Lytic reactivation of EBV alters m^6^A Modification of TYK2 and DTX4 mRNA **A**, **B**. Coverage plot of MeRIP (color) and input (black) reads in TYK2 and DTX4 transcripts in Akata and LCL cells during latency and reactivation, as determined by MeRIP-seq. Average normalized plot from the replicate experiments were represented. The gray shades represent the standard deviation of the duplicate samples. The dotted lines and black arrows represent regions on TYK2 and DTX4 gene that were hypermethylated during reactivation. Red arrow represents the regions that were hypermethylated during reactivation and are having the RRACH motif. **C**. MeRIP-rtPCR analysis of relative m^6^A level of TYK2 and DTX4 in latent and reactivated LCL and Akata cells. Fold enrichment was determined by calculating the fold change of IP to input Ct values. IgG precipitated RNA enrichment was used as the control. **D**. RNA expression of TYK2 and DTX4 during latency and reactivation and normalized based on GAPDH. **E**. Alteration of protein expression of TYK2 and DTX4 during reactivation in LCL and Akata cells. Reactivation was confirmed by expression of BZLF1 and gp350. Experiments were independently repeated three times, and results are presented as mean+/-s.d. from the three experiments. “***” represents p-value < 0.001; “**” represents p-value < 0.01; “*“represents p-value < 0.05 and “ns” represents no significance

We focused on two specific mRNAs that were found to be hyper-methylated during reactivation as they were both linked to the IFN signaling pathway. The Deltex E3 Ubiquitin Ligase 4 (DTX4) is related to pathways like NOTCH2 Activation, and transmission of signals to the nucleus and cytosolic sensors of pathogen-associated DNA [42]. DTX4 targets TANK-binding kinase 1 (TBK1) and leads to its ubiquitin-mediated proteosomal degradation [26]. TBK1 is responsible for the phosphorylation of IRF3 that results in its translocation to the nucleus and activation of IFN responsive genes, thus promoting interferon signaling [43]. Similarly, tyrosine kinase 2 (TYK2), interacts with the cytoplasmic domain of interferon receptors and leads to phosphorylation of downstream molecules for activation of the Interferon response [44]. During lytic reactivation we observed specific regions of hypermethylation in both the DTX4 and TYK2 mRNAs (Fig. 2A, B), as evident from MeRIP-seq data analysis. Most of the sites that were enriched with hypermethylation during reactivation also coincided with the RRACH motif (red arrowheads) which are reported as the most probable m^6^A methylation sites on RNA [45]. We validated our findings by MeRIP-PCR from LCL and Akata cells uninduced and induced for reactivation of the virus (Fig. 2C). In both LCL and Akata we saw a significant increase in TYK2 about 3-fold, and DTX4 between 5 and 40-fold. We also performed a MeRIP-PCR on latent and reactivated BL41-B95.8 cells and confirmed our previous findings in LCLs and Akata cells (Suppl. Figure 2A, B). Reactivation was also induced by treatment of EBV positive cells with anti-human immunoglobulins (IgG). We treated BL41-B95.8 EBV positive cells with anti-human IgG for 36 h to induce reactivation and found hyper-methylation of TYK2 and DTX4 mRNAs latent infection (Suppl. Figure 2A, B). This data clearly demonstrated that hypermethylation of these mRNAs are independent of the process by which cells are reactivated. We also monitored the relative mRNA levels of DTX4 and TYK2 by amplification from the total cDNA (Fig. 2D) and showed a distinctly different expression pattern in LCL and Akata when compared to the pattern for MeRIP-PCR (Fig. 2C). We observed an increase in TYK2 mRNA expression in reactivated LCL and in reactivated Akata cells. We also noticed either a small reduction or no significant change of the DTX4 mRNA levels during reactivation in LCL and Akata cells, respectively. (Fig. 2D). We further checked the level of expression of these proteins during latency and upon reactivation. Western blot data showed that TYK2 was downregulated while DTX4 was upregulated during reactivation of both LCL and Akata cells (Fig. 2E). To confirm reactivation, we also monitored the levels of BZLF1 (immediate early transactivator) and gp350 (late protein) (Fig. 2E). These data clearly indicated that expression of DTX4 and TYK2 at the protein level was independent of the levels of their specific mRNAs, and therefore the changes in protein levels observed are because they are post-transcriptionally regulated.

To identify the link between hypermethylation of mRNAs and expression of TYK2 and DTX4 at the protein levels, we expressed their cDNAs from a heterologous promoter, and knocked down the m^6^A writer METTL14 independently in BJAB cells and monitored the levels of TYK2 and DTX4. We showed that expression of METTL14 upregulated DTX4, and downregulated TYK2 levels. The reverse trends were observed when METTL14 was silenced using an shRNA METTL14 stable BJAB cell line (Suppl. Figure 2 C). To further confirm the effect of over-expression and silencing of METTL14 on methylation of TYK2 and DTX4 mRNA, MeRIP-rtPCR was performed. We observed that over-expression of METTl14 led to higher fold enrichment while silencing resulted in lower enrichment of TYK2 and DTX4 mRNA (Suppl. Figure 2D). These data strongly support a role for m^6^A methylation in regulation of TYK2 and DTX4 expression.

### m^6^A modification of TYK2 and DTX4 controls the mRNA metabolism

**Fig. 3:**
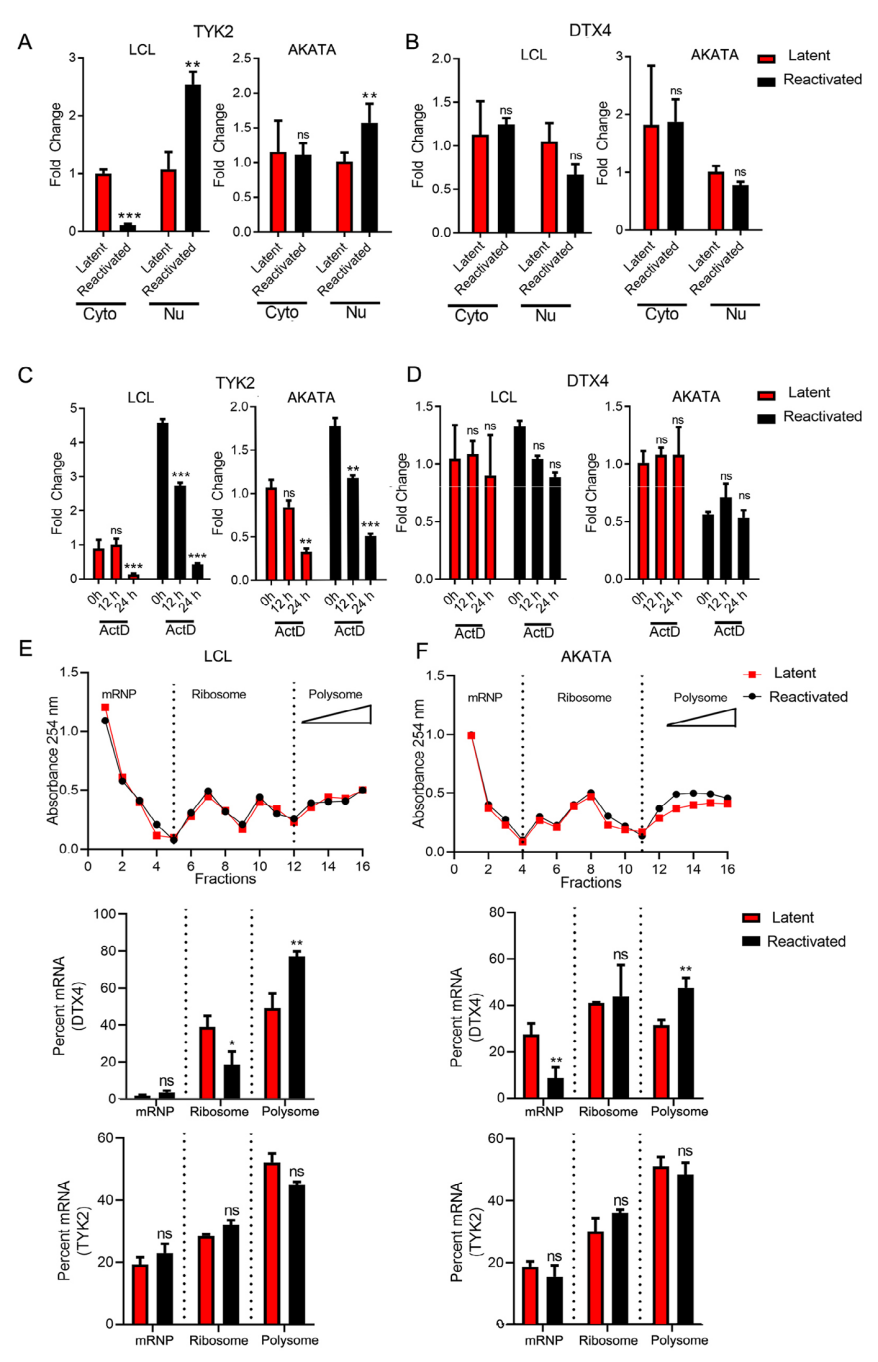
m^6^A methylation alters metabolism of TYK2 and DTX4 mRNA **A, B**. The rtPCR quantification of relative DTX4 and TYK2 RNA in nuclear and cytoplasmic fractions in latent and reactivated LCL and Akata cells. **C, D**. Measurement of TYK2 and DTX4 mRNA in latent and reactivated LCL and Akata cells. At 12, 24 and 36 h post reactivation, cell culture media was replaced with media containing Actinomycin D (ActD). RNA was harvested from cells at 36 h post-reactivation subjected to rtPCR to determine remaining relative TYK2 and DTX4 mRNA levels. **E, F**. Relative absorbance values of fractions 1–16 retrieved from ultracentrifugation of latent and reactivated LCL and Akata cell extracts over 15–50% sucrose gradients (top panel). Percent of mRNA in each set of fractions. Messenger Ribonucleoprotein (mRNP), 80S Ribosome fraction and polysome and polyribosome fraction (middle and bottom panel). Experiments were independently repeated three times, and results are presented as mean ± s.d. from the three experiments. “***” represents p-value < 0.001; “**” represents p-value < 0.01; “*“represents p-value < 0.05 and “ns” represents no significance

We next investigated the function of m^6^A in the context of the metabolism of specific mRNAs. m^6^A modification has been linked to a range of cellular processes including RNA export from the nucleus, stability, degradation, and altered ribosomal binding [1]. These processes eventually result in altered metabolism of the mRNAs and the related protein expression. We found that there was significantly less TYK2 mRNA in the cytoplasmic fractions in LCL cells that were reactivated, and no significant change in the Akata cells that were reactivated (Fig. 3A). However, in nuclear fractions in both reactivated LCLs and Akata cells, the TYK2 mRNA was significantly higher whereas no significant change was observed for DTX4 during latency and reactivation (Fig. 3B). Less mRNAs in the cytoplasmic fraction may have resulted from its degradation in the cytosol or due to altered efficiency in the export of the mRNAs from the nucleus to cytosol. To determine whether m^6^A modification has any effect on DTX4 and TYK2 mRNA stability, we treated latent and reactivated LCL and Akata cells with Actinomycin D (ActD) for different time points. ActD is a potent transcriptional inhibitor that shuts down formation of full-length mRNA transcripts. Our results showed that TYK2 mRNAs levels were significantly decreased in latent cells by 24 h but in reactivated cells the decrease was significant by 12 h treatment for both cell lines (Fig. 3C). No significant changes in DTX4 mRNA levels during ActD treatment in latent and reactivated cells were observed in either LCL or Akata cells (Fig. 3D). The decrease in the TYK2 mRNA levels in cytosol (Fig. 3A) during reactivation and the reduced mRNA stability during ActD treatment (Fig. 3C) led to the conclusion that the TYK2 RNAs may be targeted for degradation in the cytosol during reactivation.

We next investigated whether the m^6^A modification had any effect on the ribosomal binding to the mRNAs. We performed a density gradient centrifugation and isolated mRNAs from different fractions of the ribosome preparations followed by rtPCR to monitor the levels of TYK2 and DTX4 mRNAs in the different fractions. We prepared 16 fractions and grouped them into three groups based on messenger ribonucleoprotein (mRNP), 80S ribosomes, and the polysomes (Fig. 3E, F) as determined by absorbance. During reactivation in both LCL and Akata we observed significantly higher DTX4 mRNAs in the polysome fractions, as compared to the latent cells, wheras no significant changes were seen for the TYK2 mRNAs (Fig. 3E, F). Notably, mRNAs bound to polysomes have a greater efficiency of translation which leads to higher protein expression [46].

The results of these experiments led us to conclude that there was an increase in levels of DTX4 mRNA in the polysome fraction. This is congruent with our previous findings above that showed higher DTX4 protein levels during lytic reactivation (Fig. 2D).

### Hypermethylation of DTX4 mRNA induces polysome formation

**Fig. 4:**
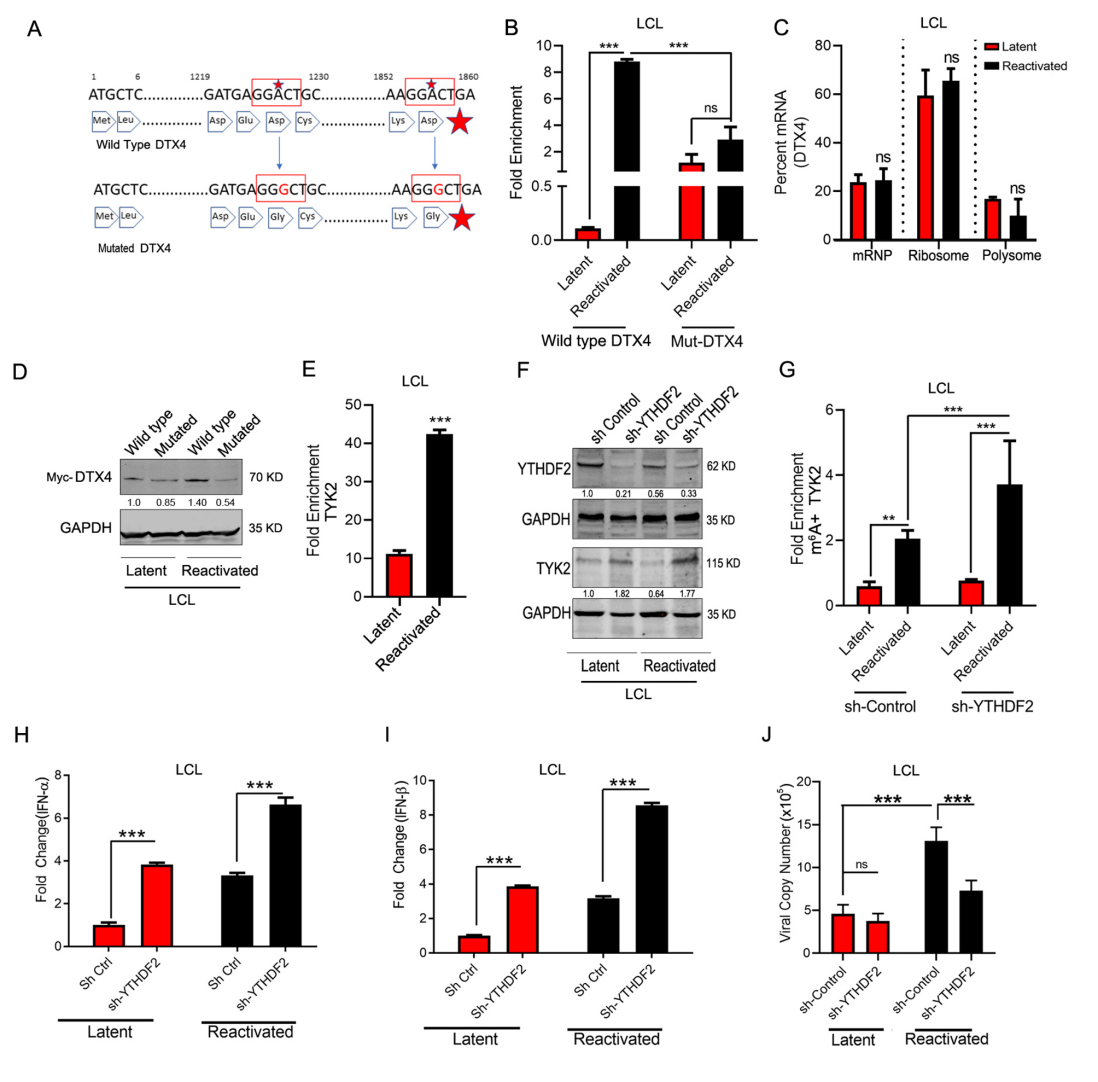
m^6^A methylation alters protein expression of TYK2 and DTX4 (**A**) Schematic of the DTX4 cDNA seq with the two specific point mutation scheme for altering A to G residues (red) that disrupt the m^6^A motifs. (**B**) RIP-rtPCR analysis of relative m^6^A level in DTX4 mRNAs in latent and reactivated LCL1 cells transfected with either mutated myc-DTX4 or wildtype myc-DTX4 control (**C**) Polysome profiling of the mutated myc tagged DTX4 transfected LCL cells followed by rtPCR of each set for finding percent of DTX4 mRNA. (**D**) Effect of the mutations on myc-DTX4 protein expression. (**E**) RIP-rtPCR analysis of relative YTHDF2 bound TYK2 mRNAs in latent and reactivated LCL1 cells (**F**) Protein expression of YTHDF2 and TYK2 in YTHDF2 knockdown latent and reactivated LCL1 cells. (**G**) RIP-rtPCR analysis of relative m^6^A level in TYK2 mRNAs in actinomycinD treated latent and reactivated LCL1 cells transfected with either sh-YTHDF2 or empty vector control **H, I**. mRNA expression of IFN-α and β by rtPCR in YTHDF2 knock down LCL1 cells during latency and reactivation. **J**. Effect of YTHDF2 knock down viral copy numbers per milliliter during latency and reactivation of LCL1 cells. Experiments were independently repeated three times, and results are presented as mean+/-s.d. from the three experiments. “***” represents p-value < 0.001; “**” represents p-value < 0.01; “*“represents p-value < 0.05 and “ns” represents no significance

We showed that m^6^A modified mRNAs were associated with polyribosome formation, and predicted different motifs targeted for m^6^A modification using an online program SRAMP [45], on the DTX4 gene. The distribution of m^6^A on mRNAs is specific and is reported that the methylated adenosine is located in RRACH or DRACH motif (R = A or G, H = A, C or T, D = A, G or T) [47]. We selected two motifs that were most probable targets for modification. We inserted point mutations that modified the motifs from GGACT to GGGCT within the DTX4 cDNA expression plasmid in LCL (Fig. 4A). We performed immunoprecipitation for m^6^A methylation followed by rtPCR for DTX4 . We observed significant reduction of fold enrichment in the mutated reactivated cells compared to the reactivated control cells exogenously expressing wild type DTX4 (Fig. 4B). To further verify whether these two specific regions are methylated, and if point mutations can disrupt the motifs leading to the removal of the methylation marks on these regions, we did RIP-rtPCR with primers specific for these regions (Primer Set1 and Primer Set2). LCL1 cells with either wild type DTX4 (WT-DTX4) or with mutated DTX4 (Mut-DTX4) were reactivated and RNA immune precipitation was performed with antibodies specific for m^6^A methylation and did rtPCR using these specific sets of primers. Our result shows that mutations significantly reduced m^6^A enriched RNA as compared to the wild type for Primer Set 1 (Suppl. Figure 3 A) while Primer Set 2 showed relatively less change (Suppl. Figure 3B). This suggests that the region which Primer Set 1 (1693–1860 nucleotides) amplified had more methylation compared to the region amplified by Primer Set 2 (1210–1456 nucleotides). This clearly demonstrated that mutation of the RRACH motif in the DTX4 gene led to a significant reduction of the methylation of the DTX4 mRNAs. To further confirm the effect of this reduced methylation, we performed polysome profiling and found that disruption of this motif significantly altered DTX4 mRNA levels within the polysome fraction. We earlier showed a higher percentage of DTX4 mRNA in the polysome fraction of reactivated cells (Fig. 3E, F).However in cells having the mutated DTX4 m^6^A motif there was no significant difference between the latent and reactivated cells (Fig. 4C). We further confirmed the expression of myc-DTX4 at the protein level in LCL cells (Fig. 4D). The levels of myc-DTX4 increased during reactivation in cells expressing the wild type DTX4. Moreover, we noticed that during reactivation the levels of mutated myc-DTX4 was significantly less as compared to the levels of cells expressing wild type myc-DTX4 (Fig. 4D). We concluded that during EBV reactivation DTX4 mRNAs were hypermethylated for m^6^A which facilitates its association with polysomes, and that this subsequently led to an increase in protein level.

### YTHDF2 mediated degradation of hypermethylated TYK2

The m^6^A methylated sites on the RNAs are recognized by the reader proteins that belongs to a specific YT521-B Homology Domain-containing Family (YTHDF) family of m^6^A-binding proteins. The YTHDF family has three paralogs (DF1, DF2 and DF3) and each of which has different reported functions [48]. YTHDF2 is the reader protein that recognizes the m^6^A methylation on mRNAs and regulates their stability by inducing their degradation through interaction with the complex of CCR4-NOT and P/MRP [49, 50]. We previously showed altered stability of the TYK2 mRNAs during reactivation (Fig. 3C). We then performed RIP using antibodies against the YTHDF2 protein followed by rtPCR for TYK2 transcripts in LCL cells. We found a significant increase in fold enrichment of TYK2 transcripts in reactivated cells when compared to the latently infected LCL cells (Fig. 4E). The data strongly suggests that a greater amount of TYK2 mRNAs were bound to YTHDF2 during reactivation compared to latently infected cells. To further determine the involvement of YTHDF2 in degradation of TYK2 mRNA, we monitored the protein levels of TYK2 after silencing YTHDF2 in both latent infection and after reactivation. Our data clearly shows that when YTHDF2 was knockdown using an YTHDF2 specific shRNA lentivirus in LCL, the levels of TYK2 were rescued in both latent and reactivated cells (Fig. 4F). We further treated the YTHDF2 knockdown cells with Actinomycin D and reactivated them. RNAs were immunoprecipitated using m^6^A specific antibodies which was followed by rtPCR using TYK2 specific primers. We observed significantly higher fold enrichment during reactivation in the TYK2 knockdown cells compared to the sh-control cells (Fig. 4G). This finding indicated that the TYK2 mRNAs were stabilized and accumulated after YTHDF2 knockdown. These results strongly suggests that YTHDF2 is a critical regulatory molecule responsible for stability and degradation of TYK2 mRNA.

### Direct involvement of TYK2 and DTX4 in IFN signaling during latency and reactivation

To demonstrate the direct involvement of TYK2 and DTX4 in regulation of the IFN signaling pathway, we silenced both TYK2 and DTX4 in LCL cells using shRNA specific lentivirus constructs (Suppl. Figure 4 A) and analyzed the levels of phosphorylated STAT1, 2 and IRF3. We further quantified the IFN-α and β mRNAs in these KD cells. We observed a significant decrease in the phosphorylation of STAT1 and STAT2 in TYK2 cells while increased p-IRF3 in the DTX4 KD cells (Suppl. Figure 4B, C). Using rtPCR analysis for detection of the transcripts we showed that on reactivation, the silencing of DTX4 led to an upregulation of IFN-α and β while silencing TYK2 had the opposite effect (Suppl. Figure 4D, E). As TYK2 is one of the key intermediate effector molecules of the IFN signaling pathway, and IFN signaling has a direct impact on the viral genome copy numbers during reactivation, we determined the mRNA levels of IFN-α and IFN-β (Fig. 4H, I). The results showed that IFN-α and IFN-β levels increased in both latent and reactivated cells when YTHDF2 was knocked down between 2 and 4- fold. This significant upregulation of the IFN-α and IFN-β levels also correlated with a subsequent decrease in the viral copy number when YTHDF2 was silenced (Fig. 4J). Previous studies suggest that viral infection can lead to hypermethylation of IFN-β mRNA which can lead to its degradation [8]. To demonstrate whether reactivation can directly degrade IFN-β mRNAs through methylation, we reactivated the sh-control cells and sh-YTHDF2 cells and after 12 h of reactivation, 1 mM of ActD was treated and incubated for another 12 h. Total RNA was isolated and the levels of IFNB was determined using specific primers. We found treatment with ActD for 12 h had no significant effect on levels of total IFN-β mRNA. This finding concludes that during EBV reactivation the IFN-β mRNAs are not significantly degraded through direct m6A methylation (Suppl. Figure 4 F).

These results strongly support our conclusion that hypermethylation of TYK2 mRNA during reactivation enhances its binding of YTHDF2 leading to degradation of its mRNAs and a dampened IFN response to enhance viral lytic replication.

### ALKBH5 a key modulator of m^6^A methylation for TYK2 and DTX4

**Fig. 5:**
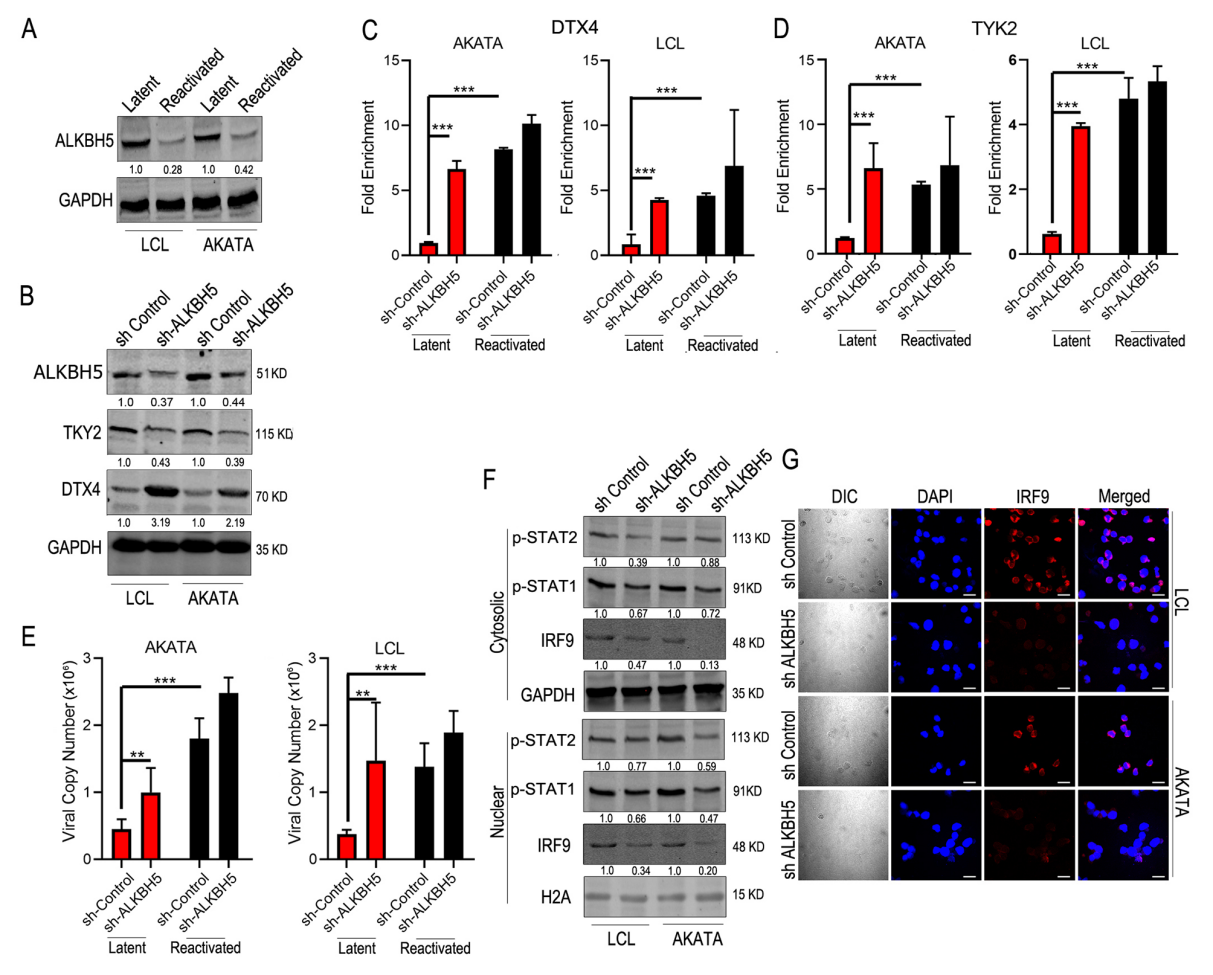
Effect of ALKBH5 on the Interferon signaling (**A**) Protein expression of ALKBH5 in latent and reactivated LCL and Akata cells. (**B**) Effect of knockdown of ALKBH5 on TYK2 and DTX4 protein levels in LCL and Akata cells. **C, D**. MeRIP-rtPCR analysis of relative m^6^A level of TYK2 and DTX4 in knock down ALKBH5 condition of latent and reactivated LCL and Akata cells. **E**. Effect of knockdown of ALKBH5 on viral copy numbers per milliliter during latency and reactivation. **F**. Alteration of levels of IRF9 and STAT protein expression in nuclear and cytosolic fraction of ALKBH5 knock down cells. **G**. Microscopic analysis of the expression of IRF9 in LCL and AKATA cells during ALKBH5 knock down conditions. Experiments were independently repeated three times, and results are presented as mean+/-s.d. from the three experiments. “***” represents p-value < 0.001; “**” represents p-value < 0.01; “*“represents p-value < 0.05 and “ns” represents no significance

Previous studies showed that during lytic reactivation of EBV, the m^6^A writer complexes were downregulated [28]. However, in the present study we found that during lytic reactivation a number of cellular transcripts were hypermethylated. We therefore investigated the concentration of the eraser molecule ALKBH5. Our findings showed that the ALKBH5 eraser was significantly downregulated during reactivation (Fig. 5A), which aligns with previous studies [28]. To determine the involvement of ALKBH5 in EBV reactivation we knocked down ALKBH5 in LCL and Akata cells and compared the levels of TYK2 and DTX4 protein with that of control cells. We confirmed the KD of ALKBH5 by western blot (Fig. 5B). Western blot analysis showed a significant downregulation of TYK2 (> 2-fold) and upregulation of DTX4 (2-3-fold) in knockdown cells compared to the sh-control during latency (Fig. 5B). We also performed MeRIP with m^6^A methylation followed by rtPCR in cells with ALKBH5 silenced. The results showed that on silencing ALKBH5 in latent cells there is a significantly higher-fold enrichment of methylation in both TYK2 and DTX4 mRNAs (Fig. 5C, D), and is consistent in both LCL and Akata cells. This data strongly showed that ALKBH5 was responsible for removal of the methylation marks during latency. We can therefore infer that during latency there is higher expression of the writer complex (METTL3/METTL14). However, as the activity level of the eraser molecule (ALKBH5) is also high, the m^6^A methylation marks were being removed. During lytic reactivation, the concentrations of both the writer and the eraser complex decreased, which led to retention of the m^6^A methylation marks on some of the mRNA transcripts. Therefore we were able to detect hypermethylation of some of the transcripts during reactivation despite having lower levels of writer complexes.

Lytic reactivation is a complex series of events that involve attenuation of the cellular immune responses with the resulting production of progeny viruses. We showed that on silencing ALKBH5 there was a significant increase in viral copy numbers even during latent infection (Fig. 5E). We also demonstrated a significant decrease in the IFN-α/β levels in sh-ALKBH5 KD LCL cells compared to the sh-control during reactivation (Suppl. Figure 5 A). To further probe the downstream signaling activities of the IFN pathway when ALKBH5 was silenced we monitored IRF9, STAT1 and STAT2 levels in cytoplasmic and nuclear fractions. The results showed lower phosphorylation levels of STAT1 and 2 (Fig. 5F), and a significant decrease in IRF9 expression levels by western blot and immunofluorescence signals (Fig. 5F and G). We also monitored several known Interferon regulated genes. Five of these genes (IRF7, NOS2, ISG15, Viperin, IRF1) were dramatically downregulated when ALKBH5 was silenced (Suppl. Figure 5B). However, one gene IFI27, a known antiviral response gene [51], did not show any noticeable change in LCL (Suppl. Figure 5B). Nevertheless, it was reduced about 2-fold in Akata cells (Suppl. Figure 5B). These findings clearly support the role for ALKBH5 in suppression of the IFN response during viral reactivation.

## Discussion

Methylation of the nascent RNA is one of the most abundant RNA modifications responsible for changes in RNA metabolism. Altered RNA metabolism not only involves RNA turnover, but also affects other processes like RNA transport, ribosome binding and alternative splicing. Therefore, RNA methylation can directly impact the expression of the specific proteins modified [52]. Studies have shown that the m^6^A modifications regulate innate immune responses and can play a crucial role in a range of pathophysiological conditions including host pathogen interactions and auto-immune disorders [8]. Recent studies on host pathogen interactions reveal that viral infection alters the host m^6^A methylation landscape [37]. These alterations provide an additional advantage to the virus over the host to establish a successful infection.

The innate immune response plays an important role in the biology of viral infections [53]. Innate immune sensor molecules recognize viral nucleic acids (RNA or DNA), and trigger IFN signaling, which involves the JAK-STAT pathway, and in turn activates the IFN responsive genes to initiate the cellular clearance process that also includes inhibition of viral replication [12, 54, 55]. Secreted interferons function in both autocrine and paracrine fashion [56, 57], and triggers the expression of Interferon stimulated genes (ISGs) [58], not only in the infected cells but also in uninfected cells in the microenvironment. This leads to a robust antiviral response.

Gammaherpesviruses, especially EBV after infection effectively establishes latency within a few days with restricted gene expression [19]. During latency, the virus expresses a small subset of latent antigens [19]. In addition, physiological stress once sensed by the virus leads to expression of immediate early genes and subsequently the late genes, which when expressed lead to a successful lytic reactivation with the production of progeny virus. During the reactivation phase, the virus replicates and progeny viruses are produced. At the same time the host system also recognizes the foreign DNA and initiates its antiviral response. Therefore, to successfully complete reactivation the virus must subvert the host antiviral response [40]. There are numerous studies on different mechanisms employed by EBV to manipulate the innate immune response to create a more favorable environment for production of progeny virions [40]. Our study is the first to comprehensively link how EBV can manipulate the host m^6^A modification system during reactivation to suppress the host innate immune response that will result in successful completion of lytic reactivation.

The N6-methyladenosine (m^6^A) methyl transferase system is composed of three different complexes namely the writer, reader and the eraser complex [59]. The writer complex, is a heteromeric complex of METTL3, METTL14, VIRMA, RBM and WTAP [60] that recognizes specific motifs on the mRNA, and methylates the adenosine residues within the motif [61]. The reader complex is made up of the YTH domain family members, YTHDF1, 2, 3 or insulin-like growth factor 2 mRNA-binding proteins (IGF2BPs)[62]. The specific reader protein that is bound to the methylated adenosine determines the fate of the mRNA. Among these reader molecules, YTHDF2 is the one predominantly associated with the degradation of the mRNA [63]. The eraser is the heterodimer ALKBH5/FTO complex, which removes the methyl group from the adenosine [64]. Previous studies have shown that alteration in expression of these molecules is associated with a number of pathophysiological conditions [65, 66]. Our lab has previously shown that during latency, the EBV-encoded nuclear latent antigen 3 C (EBNA3C) interacts with the METTL14 and is responsible for upregulation of the overall methylation of the viral transcripts [28].

In the present study, we investigated the status of the host epitranscriptome during lytic reactivation of EBV. We identified 7 genes that were differentially methylated during reactivation (Fig. 1D, E), among which two genes namely DTX4 and TYK2 were both hypermethylated during reactivation and are key members of the IFN signaling pathway. We therefore examined the expression of these genes and confirmed that during reactivation TYK2 was downregulated while DTX4 was upregulated at the protein level (Fig. 2E). We demonstrated that hypermethylation on DTX4 upregulates its expression while the opposite for TYK2 expression. To elucidate the exact role of m^6^A methylation on these two mRNAs transcripts, we performed a series of experiments. First, we looked into whether methylation had any effect on the transport of mRNA transport from nucleus to cytosol. Our data clearly demonstrated that m^6^A methylation imparts an effect on the stability of TYK2 mRNA (Fig. 3A, C). Polysome profiling revealed that increased methylation led to higher DTX4 mRNA in the polysome fraction during reactivation suggesting that this transcript is more efficiently translated (Fig. 3E, F). This also indicated that DTX4 had increased translational efficiency during reactivation. These finding supports our initial finding of higher DTX4 protein levels during reactivation compared to latency. To further confirm the involvement of m^6^A methylation in alteration of DTX4 protein levels during reactivation, we cloned DTX4 cDNA and introduced two point mutations within the gene that disrupts the m6A motifs (Fig. 4A). We detected a considerable drop in m^6^A enriched DTX4 mRNA during reactivation as compared to the non-mutated vector control (Fig. 4B). Polysome-profiling of the mutated cells (Fig. 4C) revealed that on disruption of the RRACH motif, there was significantly less DTX4 mRNA in the polysome fraction compared to wild type. Notably, we saw less DTX4 expression as compared to the wild type during reactivation suggesting that m^6^A modification of DTX4 influences its protein expression. We then examined the stability of TYK2 mRNA and found that during reactivation its mRNAs were less stable. RIP with YTHDF2 specific antibody followed by rtPCR using specific primers for TYK2 revealed that YTHDF2 was bound at greater quantities to the TYK2 mRNA during lytic reactivation as compared to the latent transcripts. We further validated the role of YTHDF2 by knockdown of YTHDF2 and observed that TYK2 levels were restored during reactivation. We also observed higher fold enriched m^6^A methylated TYK2 mRNAs in YTHDF2 KD cells during reactivation. These findings clearly demonstrated the importance of YTHDF2 in regulation of TYK2 mRNA stability and hence its expression. Since TYK2 is an important member of the IFN signaling pathway, we examined the effect of Knockdown of YTHDF2 on the IFN response, and on viral copy numbers during reactivation. Silencing of YTHDF2 not only triggered the IFN-α/β production, but also led to significant decrease in viral copy numbers during reactivation. Interestingly, during latency YTHDF2 knockdown cells had a significantly higher amount of IFN-α/β but no significant change in the viral copy numbers. We speculate that during latency LCL cells harbors a very small number of viral episomes, and so even an increase in the IFN response would have little to no change in the viral copy numbers. We also observed a similar effect when we silenced TYK2. However, silencing of DTX4 had an inverse effect. Knock down of DTX4 gene resulted in upregulation of both IFN-α, β production.

We previously showed that during reactivation, the expression of the writer complex (mainly METTL14) was downregulated [28]. However, some cellular transcripts were found to be hypermethylated during reactivation. To explore this in more detail we examined the levels of the eraser molecule (ALKBH5). Similar to our previous finding [28], ALKBH5 (the eraser molecule) levels were much higher during latency compared to the lytic reactivation. During latency, silencing of ALKBH5 led to a significantly higher m^6^A enriched TYK2 and DTX4 mRNA. During reactivation ALKBH5 was downregulated. However, knock down of ALKBH5 resulted in downregulation of TYK2 and upregulation of DTX4 at the protein levels, as well as a pronounced effect on the viral copy numbers. Further examination of known IFN pathway genes indicated that during reactivation EBV downregulated ALKBH5 levels, which resulted in accumulation of the m^6^A on TYK2 and DTX4 transcripts leading to their altered expression.

The innate antiviral response initiates through interferon signaling (mainly Type1 and Type3), which leads to activation of Type2 IFN and production of IFN-γ [67]. Activation of Type1 IFN signaling initiates with phosphorylation of STAT1 and STAT2 by Jak-TYK2, which leads to the formation of ISGF3, a hetero-complex comprised of p-STAT1, p-STAT2 and IRF9 [68]. Similarly, activation of IFNAR complex leads to formation of a homodimer complex of pSTAT1. Both ISGF3 and p-STAT1 homodimers are transported to the nucleus where they initiate the transcription of the IFN responsive genes like ISGs, NOS2, Viperin, IRFs and TRIMs [67, 69]. We also observed significantly less IRF9 and phosphorylated STAT1 and 2 in both cytoplasmic and nuclear fractions of ALKBH5 knock down LCL1 and Akata cells. Confocal microscopy data also supported the findings showing that expression of IRF9 in these cells was significantly diminished when compared to parent control cells. We also investigated six other interferon responsive genes and noticed that five genes namely ISG7, NOS2, ISG15, VIPERIN and IRF1 were all down regulated at the transcript levels. However, one gene IFI27 had no noticeable change in LCL, but changed in Akata cells, although not as dramatic.

From these findings we conclude that during lytic reactivation EBV regulates the host RNA methylation machinery which leads to hypermethylation of specific mRNA transcripts including TYK2 and DTX4. Hypermethylation of TYK2 mRNAs led to YTHDF2-mediated degradation, while hypermethylation of the DTX4 mRNAs induced polysome formation leading to upregulation at the protein level. These processes combined, subvert the IFN response and thus result in successful lytic reactivation. Therefore, we propose a novel mechanism by which EBV modulates the host cellular immune system during lytic reactivation.

## Conclusion

**Fig. 6:**
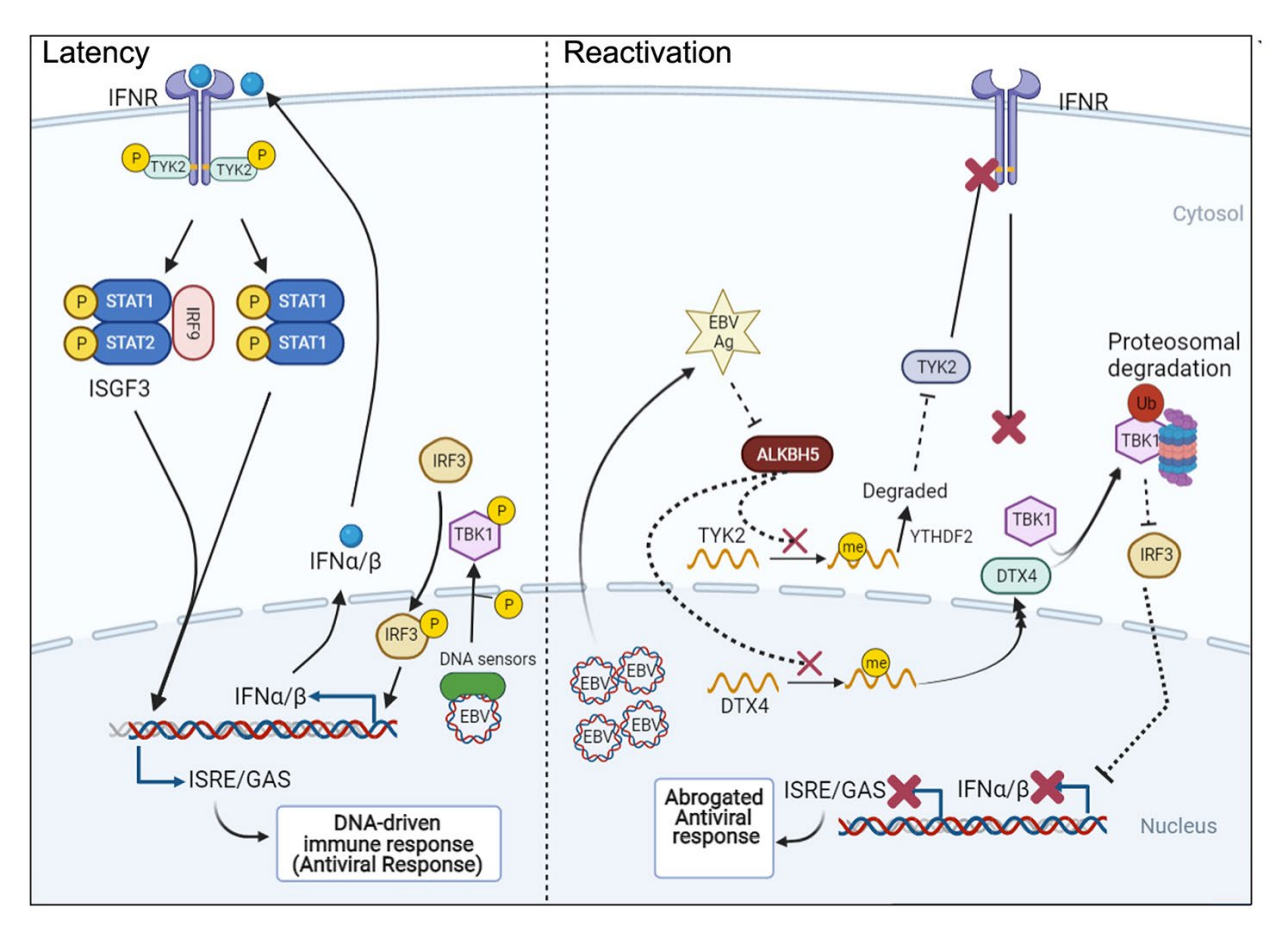
Schematic presentation of the molecular mechanism of m^6^A mediated regulation of the interferon signaling pathway in response to lytic EBV infection and reactivation Latently infected EBV cells produce a basal level of interferon that bind to the interferon receptors and trigger the downstream signaling that involves TYK2 and ISGF3 which drives the innate antiviral response. During reactivation the virus inhibits the activity of eraser molecule, ALKBH5. This leads to retention of methylation in some host transcripts including TYK2 and DTX4. Methylated TYK2 transcripts are rapidly degraded while methylated DTX4 are translocated to the cytosol to promote degradation of TBK1. These events lead to dampened IFN response and results in an abrogated antiviral response

During latency the methyl transferase m^6^A writer and eraser complexes were maintained at higher concentration in EBV latent cells. However, during reactivation, the concentration of both writer and eraser complexes drops. This leads to retention of m^6^A mark on specific cellular mRNA transcripts including DTX4 and TYK2. Hypermethylation of TYK2 mRNAs leads to increased degradation of TYK2 mRNA while hypermethylation of DTX4 results in its increased association with polyribosomes. As would be expected, these changes resulted in decreased TYK2 and increased DTX4 at the protein level. The opposing signals for these two proteins in terms of their levels were also reflected in their activities in the interferon signaling pathway. Silencing of the eraser molecule (ALKBH5) in latency led to mimicking of that seen in reactivation, and dampened IFN signaling, which resulted in an increase in viral copy number (Fig. 6). This study provides new insights into the role of m^6^A modification on regulation of EBV reactivation through control of IFN signaling. However, more is needed to be done in exploring some of the antigens involved in regulating ALKBH5 expression and whether or not this may have a broader impact on other viral systems in their control of the innate immune response through regulation of IFN signaling.

## Electronic supplementary material

Below is the link to the electronic supplementary material.

Supplementary Material 1

Supplementary Material 2

## Data Availability

The datasets (RNA seq and MeRIP-seq) generated and/or analyzed during the current study is submitted in GEO database (GSE210618).
