# Supplementary material for: Attenuation of IFN signaling due to m6A modification of the host epitranscriptome promotes EBV lytic reactivation
Source: J Biomed Sci. 2023 Mar 14;30:18. doi: 10.1186/s12929-023-00911-9 (PMC10012557; doi:10.1186/s12929-023-00911-9)
Supplement: Supplementary file 1 — Supplementary Material 1 [file 12929_2023_911_MOESM1_ESM.docx]

**Supplementary Information (SI)**

**
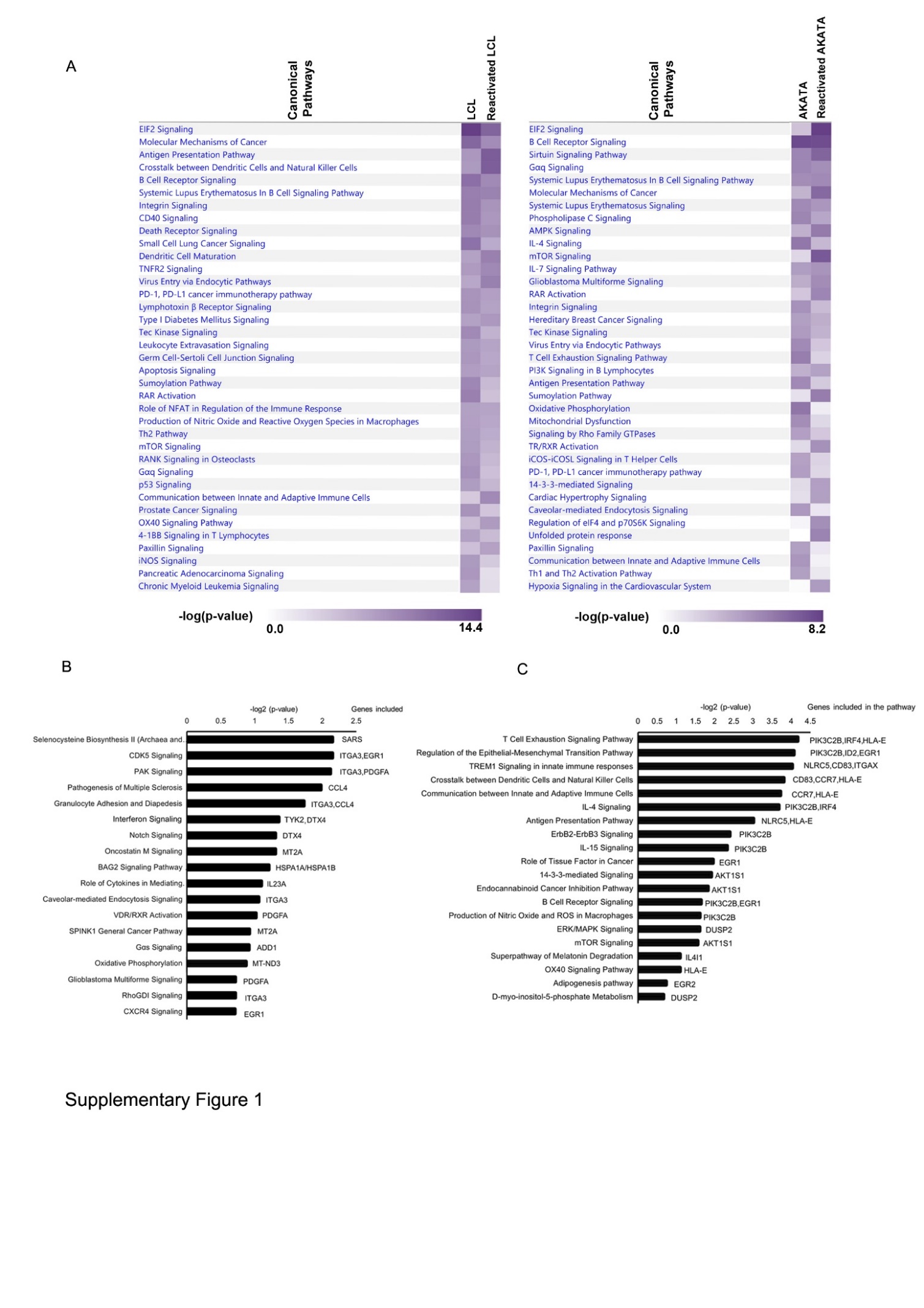
Supplementary Figures**

Supplementary Figure 1.

A. Pathway enrichment for genes with roles in different cellular processes when making comparisons between latent and reactivated LCL, and similarly between Latent and reactivated Akata cells. B. Pathway analysis of 26 genes that were differentially methylated in reactivated LCL were compared to latent LCL cells. C. Pathway analysis of 27 genes that were differentially methylated in latent Akata were compared to reactivated Akata. Each of the cellular pathways and the genes involved in these pathways may be differentially regulated in latency and lytic reactivation and are listed.


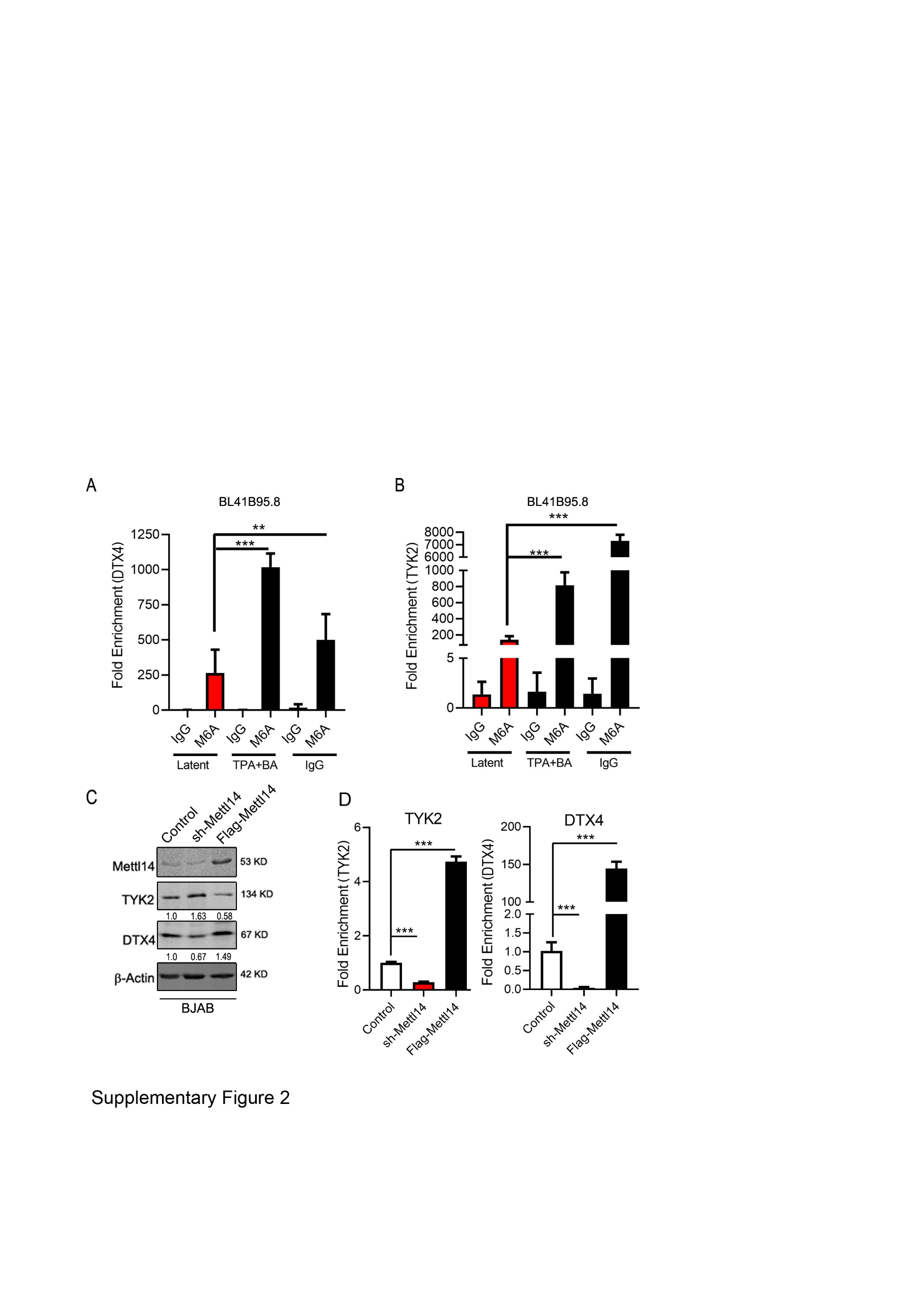
Supplementary Figure 2

A. RNA expression of TYK2 and DTX4 during latency, and reactivation of BL41-B95.8 cells. B. BL41-B95.8 cells as control untreated or reactivated when treated with TPA-Butyric acid, or by induction with anti-human IgG for 36 h. RIP-rt-PCR analysis of relative m^6^A level of TYK2 and DTX4 in latent and reactivated BL41-B95.8 cells. C. Alteration in the expression of TYK2 and DTX4 in EBV negative BJAB cells that were either knocked down for METTL14 or overexpressed METTL14 from a heterologous promoter. The band intensities were measured and normalized based on signal intensities for GAPDH. D. MeRIP-rt-PCR analysis of relative m^6^A enriched TYK2 and DTX4 mRNA in METTL14 over-expressed or knockdown BJAB cells. The fold enrichment was calculated based on RIP signal normalized based on the IgG signal. Experiments were independently repeated three times, and results are presented as mean+/-s.d. from the three experiments. “***” represents p-value <0.001; “**” represents p-value <0.01; “*“represents p-value <0.05 and “ns” represents no significance.


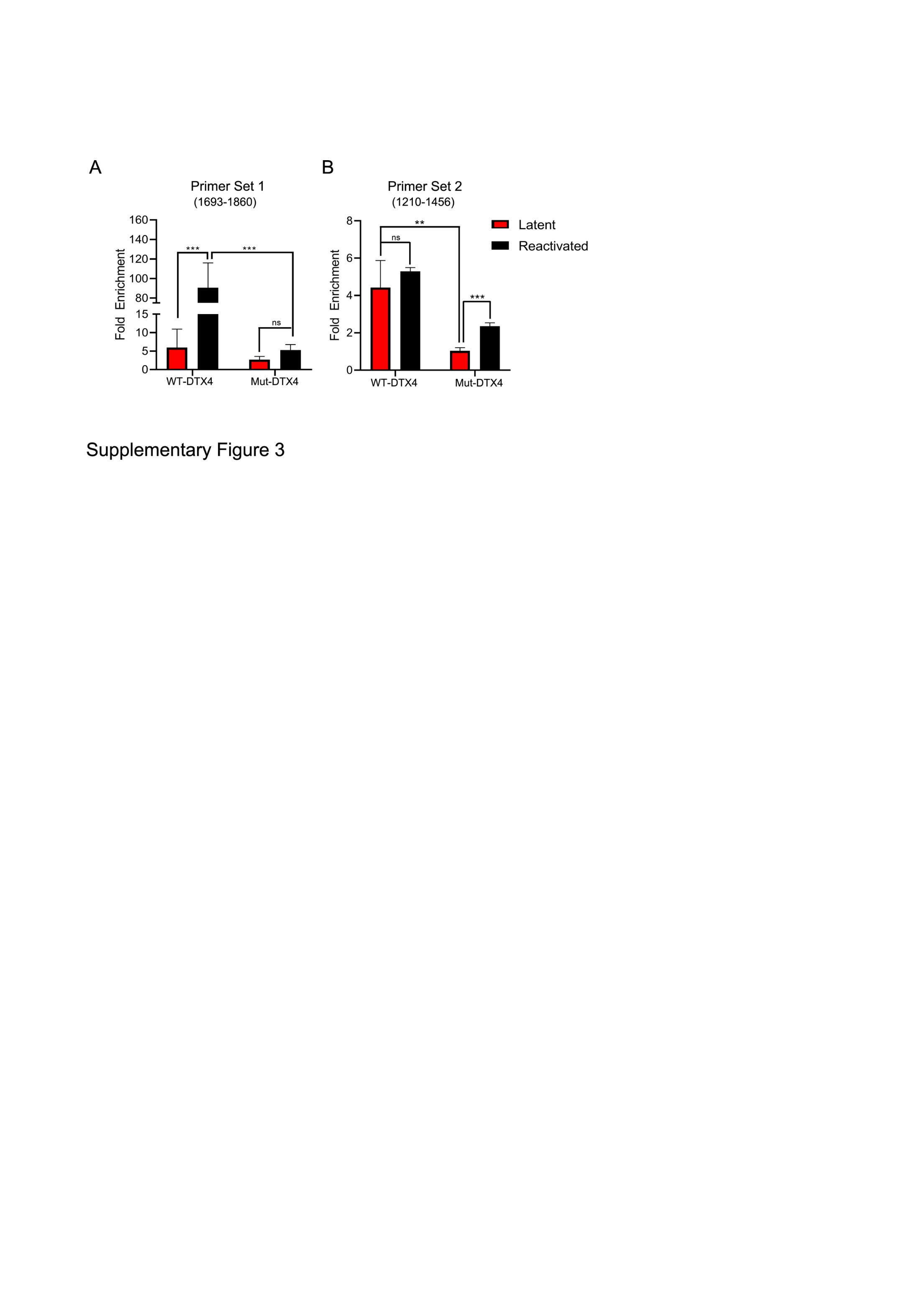


Supplementary Figure 3

LCL cells were transfected with either WT-DTX4 or with Mut-DTX4 then reactivated and RNA immunoprecipitation done using specific antibody for m^6^A. Realtime PCR was performed using specific primers for DTX4 that can amplify specific regions on the DTX4 gene. A. meRIP-rtPCR using primer set 1 (1693-1860). B. meRIP-rtPCR using primer set 1 (1210-1456).


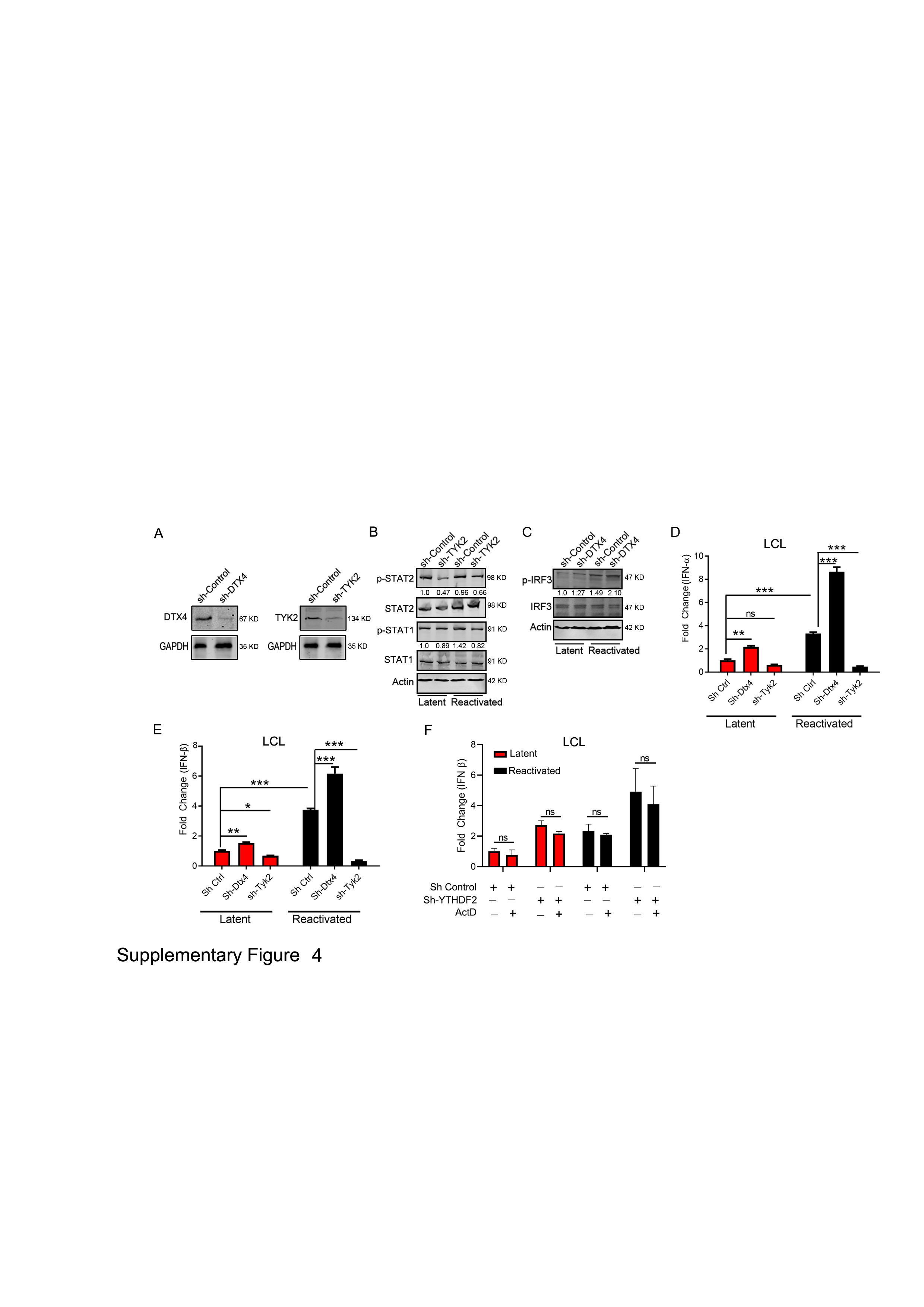
Supplementary Figure 4

A. LCL cells were transduced with short hairpin RNA designed against DTX4 and TYK2 and the expression of DTX4 and TYK2 were monitored by Western blot. B, C. Alteration in the levels of phosphorylation of Stat1, 2 and IRF3 in TYK2 and DTX4 knock down LCL cells. The band intensities were measured and normalized based on signal intensities for Actin. D, E. mRNA expression of IFN-α and β in LCL cells that were transduced with either mock or sh-DTX4 or sh-TYK2. F. Effect of Actinomycin D (ActD) treatment on LCL cells silenced for YTHDF2 during latency and lytic reactivation. Experiments were independently repeated three times, and results are presented as mean+/-s.d. from the three experiments. “***” represents p-value <0.001; “**” represents p-value <0.01; “*“represents p-value <0.05 and “ns” represents no significance.


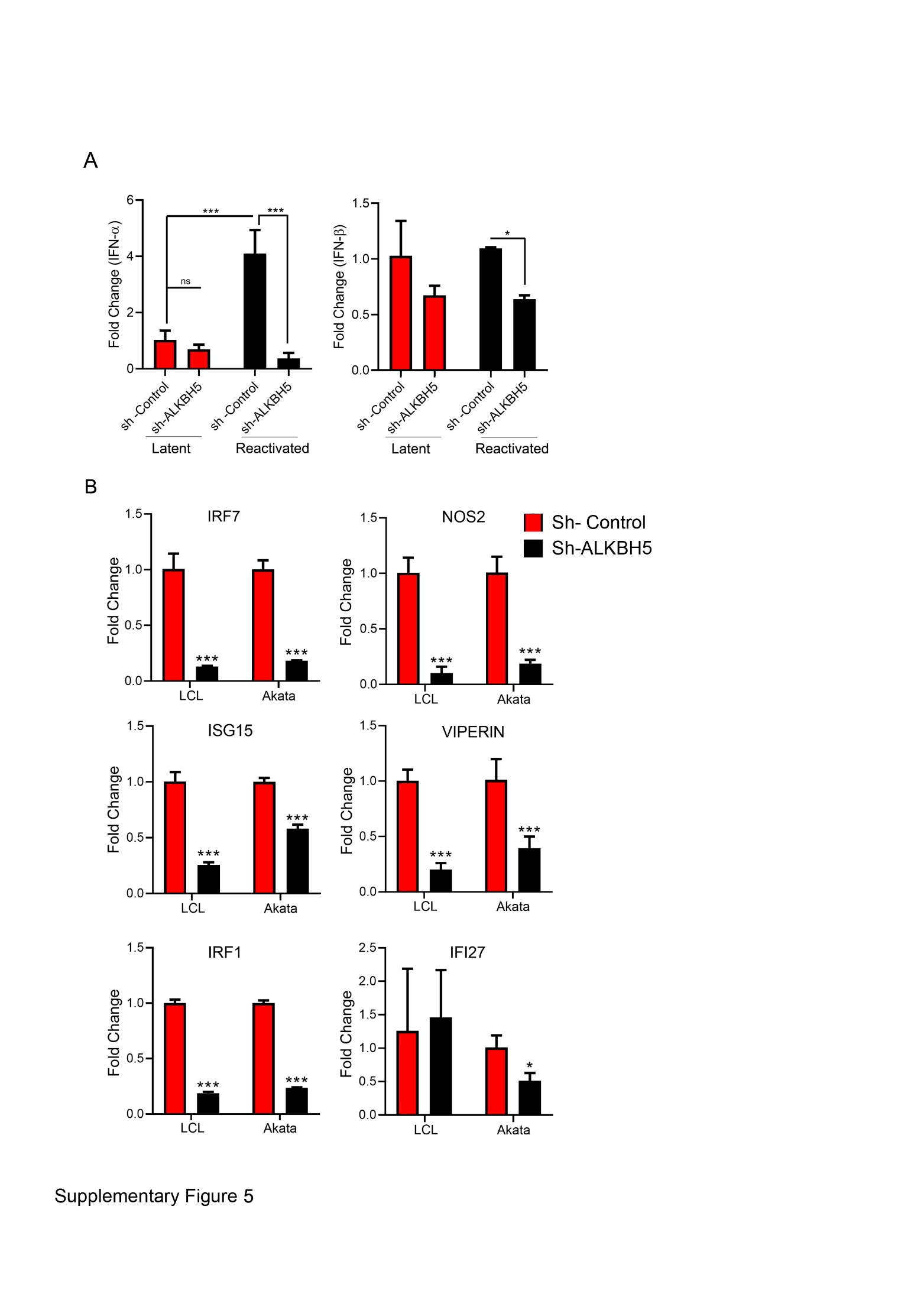


Supplementary Figure 5

Effect of Knockdown of ALKBH5 on IFN signaling in LCL and AKATA cells

A. mRNA expression of IFNα and IFNβ in LCL cells that were transduced with Lentivirus containing either mock or sh-ALKBH5. B. mRNA expression of ISG7, NOS2, ISG15, Viperin, IRF1 and IFI27 in LCL and Akata cells that were transduced with either mock or sh-ALKBH5. Experiments were independently repeated three times, and results are presented as mean+/-s.d. from the three experiments. “***” represents p-value <0.001; “**” represents p-value <0.01; “*“represents p-value <0.05 and “ns” represents no significance.

Supplementary Table 1: Cloning primers for insertion of mutation in DTX4 cDNA

| Primer Name | Sequence |
| --- | --- |
| Cloning Primer F | CCCAAGCTTGGGATGCTCCTGGCCTCGGCCGT |
| Cloning Primer R (with mutation) | GGAATTCCGCCCTTCTCCTGGGCAGTGCTG |
| Overlap primer F (with mutation) | CCGGCACCCACCAGATGAGGGCTGCACCATCTGTATGGAAC |
| Overlap primer R (with mutation) | GTTCCATACAGATGGTGCAGCCCTCATCTGGTGGGTGCCGG |

Supplementary Table 2: List of antibodies

| Antibody Name | Company | Catalog No |
| --- | --- | --- |
| Anti-N6-methyladenosine (m6A) antibody | Abcam | ab151230 |
| Anti-EBV ZEBRA Antibody (BZ1) | Santa Cruz Biotechnology | sc-53904 |
| Anti-EBV gp350 Envelope Protein Antibody (10B5) | Santa Cruz Biotechnology | sc-56981 |
| Anti-TYK2 Antibody (C-8) | Santa Cruz Biotechnology | sc-5271 |
| DTX4 Polyclonal antibody | Proteintech | 25222-1-AP |
| Anti-TBK1 Antibody (108A429) | Santa Cruz Biotechnology | sc-52957 |
| Anti-GAPDH Antibody (0411) | Santa Cruz Biotechnology | sc-47724 |
| YTHDF2 Polyclonal antibody | Proteintech | 24744-1-AP |
| Anti-Myc (9E10) | Generated from hybridomas |  |
| ALKBH5 Polyclonal antibody | Proteintech | 16837-1-AP |
| Anti-p-Stat1 Antibody (A-2) | Santa Cruz Biotechnology | sc-8394 |
| Phospho-Stat2 (Tyr690) Antibody | Cell Signaling Technologies | 4441 |
| IRF9 Polyclonal antibody | Proteintech | 14167-1-AP |
| Anti Stat1 antibody | Santa Cruz Biotechnology | sc-417 |
| Anti Stat2 antibody | Santa Cruz Biotechnology | sc-514193 |
| Anti-IRF3 | Proteintech | 11312-1-AP |
| Anti-p-IRF3 | Cell Signaling Technologies | 4947 |
| Anti Actin antibody | Santa Cruz Biotechnologies | Sc-1616 |

Supplementary Table 3: Sequences of primers and oligos.

| Primer name | Sequence |
| --- | --- |
| TYK2 F | TCTTTGAGCAGGGCAAGCAT |
| TYK2 R | GTCTTCTTGGCCACCTCCTC |
| DTX4 F | TGTGTGTGGGCGTGTGTGCATT |
| DTX4 R | TTGCATCTCCCCAGGCACAGTT |
| Viperin F | AGTGTGTTCAGGCAACCTCT |
| Viperin R | AGCCGCATTTGTAGTTGCAC |
| NOS2 F | GATCAAAAACTGGGGCAGCG |
| NOS2 R | CTCATCTGGAGGGGTAGGCT |
| ISG15 F | AGATCACCCAGAAGATCGGC |
| ISG15 R | GCTCAGAGGTTCGTCGCATT |
| IRF1 F | AAAGTCGAAGTCCAGCCGAG |
| IRF1 R | TGTTGTAGCTGGAGTCAGGG |
| IRF7 F | CCCACGCTATACCATCTACCT |
| IRF7 R | GATGTCGTCATAGAGGCTGTTG |
| EBNA1 F | GGGTGATAACCATGGACGAG |
| EBNA1 R | TATGTCTTGGCCCTGATCCT |
| GAPDH F | TGCACCACCAACTGCTTAG |
| GAPDH R | GATGCAGGGATGATGTTC |
| IFN-α F | TGGCAACCAGTTCCAGAAGG |
| IFN-α R | CCGCATTCATCAGGGGAGTT |
| IFN-β F | TCTCCTGTTGTGCTTCTCCAC |
| IFN-β R | GCCTCCCATTCAATTGCCAC |
| Sh-YTHDF2 | AAGGACGTTCCCAATAGCCAA |
| Sh-ALKBH5 | AAACAAGTACTTCTTCGGCGA |
| Primer Set1  FW (1693-1717) | ACAGGCGAGTCAGACACCGTCATCT |
| Primer Set1  RV (1836-1860) | TCAGTCCTTCTCCTGGGCAGTGCTG |
| Primer Set2  FW (1210-1234) | CCACCAGATGAGGACTGCACCATCT |
| Primer Set2  RV (1432-1456) | GGTACTCCATCTTCCCTGGAGGTTG |
