# Supplementary material for: Attenuation of IFN signaling due to m6A modification of the host epitranscriptome promotes EBV lytic reactivation
Source: J Biomed Sci. 2023 Mar 14;30:18. doi: 10.1186/s12929-023-00911-9 (PMC10012557; doi:10.1186/s12929-023-00911-9)
Supplement: Supplementary file 2 — Supplementary Material 2 [file 12929_2023_911_MOESM2_ESM.docx]

To	February 16^th^, 2023

The Editor,

Journal of Biomedical Science

Subject: Resubmission of the revised manuscript “**Attenuation of IFN signaling due to m^6^A modification of the host epitranscriptome promotes EBV lytic reactivation“** for your consideration and publication.

Dear Editor,

We would like to take the opportunity to thank you and the reviewers for the comments in regards to our manuscript entitled “**Attenuation of IFN signaling due to m^6^A modification of the host epitranscriptome promotes EBV lytic reactivation**”. We have specifically modified the manuscript and the figures based on your suggestions and comments. The queries raised by the reviewers have been completely addressed in the “response sheet” and all changes are included in the respective sections of the text and figures. We hope that these changes are satisfactory, and the revised manuscript is now acceptable for publication in “Journal of Biomedical Science”

We deeply appreciate your consideration of our manuscript and are looking forward to a positive response. If you have any further queries, please do not hesitate to contact me at the address below. Thank you very much for your time and consideration.

The point by point response to the reviewer’s queries is attached here.

With Regards,

Erle S. Robertson Ph.D.

Professor and Vice-Chair, Department of Otorhinolaryngology-Head and neck surgery

Professor of Microbiology

Perelman School of Medicine

University of Pennsylvania

Email: erle@pennmedicine.upenn.edu

Tel: 215-746-0114/0116

Fax: 215-898-9557

**Response Sheet**

**Query**

Reviewer #2: - The DTX4 m6A site issue: The issue remains that the 1123 m6A-IP peak is significantly lower than input, and it is unlikely any analysis package can fix this. However, the mutation followed by meRIP-qPCR figure is convincing. I would consider the issue resolved if the meRIP-qPCR figure is included in the manuscript (which I don't see at the moment).

**Response**

As per the suggestion we have now included the data in supplementary figure 3.

**Query**

- The IFNB issue: Again, the new ActD-treated figure does a good job of resolving the issue, yet the figure is not included in the manuscript. Please put this in (it would be alright to have it in the supplementals). Otherwise all other issues are resolved, the manuscript should be good as long as the above figures are added in.

**Response**

Thank you for the suggestions and we have incorporated the data in supplementary figures 4.

**Query**

Reviewer #4: This manuscript is worth to be published.

**Response**

Thank you.

**Query**

Reviewer #5: Journal of Biomedical Science

Manuscript Number: JBMS-D-22-01599R2

Title: Attenuation of IFN signaling due to m6A modification of the host epitranscriptome promotes EBV lytic reactivation

This manuscript illuminates the significance of m6A modification in overcoming the innate immune response during EBV reactivation. The author reports that during lytic reactivation EBV targets the RNA methylation system of the host to attenuate the innate immune response by suppressing the interferon response. This facilitates successful lytic replication of the virus. I think that experiments were well designed and well done, and the results gained could lead to conclusion. Hence, this manuscript is worth publishing in Journal of Biomedical Science.

**Response**

Thank you for appreciating our research.
